# A systematic review and integrative approach to decode the common molecular link between levodopa response and Parkinson’s disease

**DOI:** 10.1186/s12920-017-0291-0

**Published:** 2017-09-19

**Authors:** Debleena Guin, Manish Kumar Mishra, Puneet Talwar, Chitra Rawat, Suman S. Kushwaha, Shrikant Kukreti, Ritushree Kukreti

**Affiliations:** 1 Genomics and Molecular Medicine Unit, Institute of Genomics and Integrative Biology (IGIB), Council of Scientific and Industrial Research (CSIR), Mall Road, New Delhi, -110007 India; 2 Institute of Human Behaviour and Allied Sciences, Dilshad Garden, Delhi, India; 3 Department of Chemistry, Nucleic Acids Research Lab, University of Delhi (North Campus), Delhi, India; 4 Academy of Scientific & Innovative Research (AcSIR), CSIR- Institute of Genomics and Integrative Biology (CSIR-IGIB) Campus, New Delhi, India

**Keywords:** Parkinson’s disease, Levodopa, Dyskinesia, Adverse effects, Levodopa response

## Abstract

**Background:**

PD is a progressive neurodegenerative disorder commonly treated by levodopa. The findings from genetic studies on adverse effects (ADRs) and levodopa efficacy are mostly inconclusive. Here, we aim to identify predictive genetic biomarkers for levodopa response (LR) and determine common molecular link with disease susceptibility. A systematic review for LR was conducted for ADR, and drug efficacy, independently. All included articles were assessed for methodological quality on 14 parameters. GWAS of PD were also reviewed. Protein-protein interaction (PPI) analysis using STRING and functional enrichment using WebGestalt was performed to explore the common link between LR and PD.

**Results:**

From 37 candidate studies on levodopa toxicity, 18 genes were found associated, of which, CA_n_ STR 13, 14 (*DRD2*) was most significantly associated with dyskinesia, followed by rs1801133 (*MTHFR*) with hyper-homocysteinemia, and rs474559 (*HOMER1*) with hallucination. Similarly, 8 studies on efficacy resulted in 4 genes in which rs28363170, rs3836790 (*SLC6A3*) and rs4680 (*COMT*), were significant. To establish the molecular connection between LR with PD, we identified 35 genes significantly associated with PD. With 19 proteins associated with LR and 35 with PD, two independent PPI networks were constructed. Among the 67 nodes (263 edges) in LR, and 62 nodes (190 edges) in PD pathophysiology, *UBC*, *SNCA*, *FYN*, *SRC*, *CAMK2A*, and *SLC6A3* were identified as common potential candidates.

**Conclusion:**

Our study revealed the genetically significant polymorphism concerning the ADRs and levodopa efficacy. The six common genes may be used as predictive markers for therapy optimization and as putative drug target candidates.

**Electronic supplementary material:**

The online version of this article (10.1186/s12920-017-0291-0) contains supplementary material, which is available to authorized users.

## Background

Parkinson’s disease (PD) is a second most common progressive neurodegenerative disorder followed by Alzheimer’s disease [1]. It affects 1.5% of the global population over the age of 65 years [2]. Characterised by motor symptoms, like gait dysfunctioning, bradykinesia, rigidity, and resting tremors, PD has been believed to be caused due to loss of dopamine at the dopaminergic neurons in the substantia nigra pars compacta [3]. Along with the dopaminergic disruption, other non-motor dysfunctioning like depression, sleep disorder, dementia are also observed in PD patients which can be a plausible consequence of both dopaminergic and non-dopaminergic systems. Pathological confirmation is obtained by the presence of Lewy bodies- fibrillar aggregates, mostly consisting of protein alpha synuclein, in the affected neurons of the brain [4].

Levodopa (or L-Dopa), ever since its discovery, has been used as a potent anti-Parkinson’s medication and functions as symptoms alleviating therapy, by maintaining the dopamine concentration at the synapse and reduce the motor fluctuations observed in PD patients [5]. Almost 15–20% of the patients do not respond to the therapy or show adverse profiles primarily, levodopa-induced dyskinesia [6] after 5 years of therapy. Managing ADR is thus one of the most challenging aspects of PD. Carriers of specific genetic polymorphisms of drug metabolising enzymes, drug transporters, drug receptors and proteins involved in drug pathway of anti-Parkinson’s drugs may predispose to adverse reactions or altered efficacy.

Several susceptibility loci have been studied already with the familial cases of PD, like *SNCA (PARK1), LRRK2, PRKN (PARK2), PINK1 (PARK6), DJ-1 (PARK7)* [7]. However very less has been elucidated about the genetic background of the sporadic cases of PD. Neurodegenerative diseases including PD are multifactorial in nature. Mechanisms like mitochondrial dysfunction, Lewy body formation, oxidative stress, altered protein handling, and inflammatory change are considered to lead to cell dysfunction and death by apoptosis or autophagy. Ageing is one of the most studied risk factor for PD, and the biochemical changes that are a consequence of aging amplify these abnormalities in PD patients’ brain [8]. Candidate studies have pin- pointed genes like *NAT2, MAOB, GST*, mitochondrial tRNA, S18Y variant of *UCHL1, SNCA, MAPT* H1 haplotype and *LRRK2* [9]. GWA studies have identified more risk loci: *BST1, GAK, HLA-DR, ACMSD, STK39, MCCC1/LAMP3, SYT11, PARK16, FGF20,* and *GPNMB*, but with lower significance to establish a valid association for clinical management [10]. Also, since the mechanism of development and progression of PD have not been elucidated fully, current treatment options are only targeted at providing symptomatic respite. Understanding of these multiple aspects of PD may potentially reward this field of study for clinical intervention.

The aim of the present article is to summarize all the studies carried out on polymorphism-association of administration of levodopa on sporadic PD patients and its treatment outcome as ADR and the altered efficacy of the drug. We, also describe the interplay of the molecular pathways involved in the mechanism of levodopa induced ADRs, LR and the disease pathology. This is an attempt to identify the molecular targets as genes and if the polymorphisms in such genes predispose certain patient population susceptible to causing ADRs and altered efficacy. For this purpose, we perform a systematic review through several online databases, select the relevant articles on the basis of pre-defined inclusion and exclusion criteria based on the focus of our study, separately, for LR, and PD disease susceptibility. These articles are further assessed for their methodological quality and finally the data was extracted for the list of genes (and its variants) associated with the drug response and disease risk. This effort has been further elaborated using computational approaches like network modelling to rule out the systematic biases from high-throughput multiple datasets and identify if there is any molecular mechanism involved in LR and PD susceptibility that intersect each other. Such proteins can be plausible targets to minimize toxicity, elaborate the therapeutic efficacy and capture disease risk.

## Methods

All the methodologies performed in the study were drawn following the Human Genome Epidemiology Network for the systematic review of genetic association studies [11–14] and the PRISMA guidelines [15].

### Data source and search strategy

A systematic search in Medline [16] and Web of science [17] was performed using standard MeSH terms “Parkinsons’s disease”, “variant”, “Polymorphism”, “SNP”, “single nucleotide polymorphism”, “pharmacogenomics”, “response” with AND/OR Boolean operators to identify all the human studies on genetics of Parkinson’s disease and/or on drug response by anti-Parkinson drugs. Also, a check for the studies that were not identified by the previous search, of pharmacogenetic relevance from the PharmGKB database [18], were added using search term “Parkinson’s disease”. The searches were limited to human studies.

### Study selection criteria

The study selection was carried out independently in two stages by two different authors (DG and MKM) from relevant articles published up to March 9, 2016. All the articles that are reviews, commentary, erratums, editorials, technical reports, news, evaluation studies were initially screened (*n* = 173). Articles that were in duplicate (*n* = 260) and published in languages other than English (*n* = 36) were also excluded. At first, the articles were screened by titles based on relevance, as obtained by the search. Secondly, the abstracts of all primarily screened articles were retrieved and assessed according to the inclusion and exclusion criteria provided in Additional file 1: s4. Further, the articles were distinctly segregated into ADR of levodopa and efficacy of the drug. Only full text articles were included in the final study corpus. In case of disagreement regarding the screening of the articles, an independent reviewer (PT) was consulted to resolve the discrepancies. The cross-references of the finally selected articles were also searched for additional relevant articles. Further, the MeSH term for the adverse effect with ‘levodopa’ or ‘L-Dopa’ were again retrieved to double-check for any missing articles of purpose. All the baseline univariate significant allelic/genotypic associations with ADR/s and with L-dopa efficacy are reported in the Additional file 2: Table S1(8a) and Additional file 3: Table S2(8b), respectively. Similar search was performed for the drug response related articles as well.

### Data extraction and quality assessment

Data were extracted by DG and MKM and checked by PT and RK. In case of sequential or multiple publications from the same group of authors, only the recent article has been included or studies which report exclusive findings. Data extracted from each eligible publication is provided in Table 1 (complete in Additional file 2: Table S1(8a)) for ADR articles and Table 2 (complete in Additional file 3: Table S2(8b) for drug efficacy studies. Ethnicity was classified as African, Asian or Caucasian [19–23]. If the ethnicity was not reported, the source population based on the country in which the study was conducted was considered e.g. Chicago. The genetic associations were stratified by ethnicity/population to explore the inter-ethnic variations. All the populations of the subjects have been categorised into its respective super-populations based on the 1000genome project (Phase II).

**Table 1:** Characteristics of included studies for assessment of association between genetic variants and ADRs in PD

Study	Population/ ethnicity	Response criteria	Age (years)^a^	Gender		Number of samples			Type of ADR	Genes	Studied variants	p- value	OR (95% CI)	Dose^a^ (drug)	FP^a^ (years)	Score
				M	F	Total	ADR	Non ADR								
Schuh A F S et al. [64]; t	Brazilian	UPDRS, HY, MMSE	67.38 ± 10.34	105	100	205	86	119	Dyskinesia	***HOMER1***	**rs4704559**	**(GG/GA) 0.04**	**0.53 (0.29-0.98)** ^*^	200 (L)	1	13
Rieck M et al. [65]; t	Brazilian	UPDRS-IV	66.88 ± 10.80	110	98	208	90	118	Dyskinesia	***ADORA2A***	**rs2298383**	**CT-0.04**	**1.89 (1.03–3.45)**	805.14 ± 310.17 (L)	8.34 ± 4.86	13
												**TT-0.02**	**2.06 (1.10–3.82)**			
											**rs3761422**	**CC-0.02**	**3.12 (1.22-7.96)**			
												**CT-0.01**	**3.28 (1.30-8.27)**			
Strong J.A et al [23]. $	Caucasian	NR	65.3 ± 1.56 early; 69.4 ± 1.25 late	57	35	92	92	NA	Dyskinesia	***DRD2***	**14 allele**	**0.04**	**3.4 (1.1-10.4)**	NA (L, C)	5	9
											**14/15**	**0.003**	**27.2 (1.4-51.0)**			
Rieck M et.al [66]	Brazilian	UPDRS-IV	65.52 ± 9.99	102	97	199	83	116	Dyskinesia	***DRD2***	**rs2283265**	**0.05**	-	780.12 ± 308.08 (L)	8.44 ± 4.92	13
										***ANKK1***	**rs1800497**	**0.02**				
Oliveri R.L et al. [57]	Italian	UPDRS-ME, AIMS, MMSE, Hamilton	64.6 ± 9.4	53	45	98	49	49	Dyskinesia	***DRD2***	**13**	**0.02**	**0.37 (0.15- 0.89)** ^*^	25mg (C); 250mg (L)	5	13
											**14**	**0.02**	**0.25 (0.07- 0.92)** ^*^			
											**15**	**0.02**	**1.94 (1.08- 3.49)** ^*^			
											**13/16**	**0.05**	-			
											**15/16**	**0.01**	**3.88 (1.28- 11.74)**			
Gorgone G et al. [32]	Italian	HY	64.5 ± 7.7 (cases)	64	78	142	60 cases	82 control	Hyper-homocysteinemia	***MTHFR***	**rs1801133**	**<0.0001**	**2.59 (1.20-5.57)** ^*^	452.0 ± 170.0 (L)	1	12
Acuña G et al. [61] $	European	NR	NA	261	148	409	135	274	Elevated liver transaminase levels	***UGT1A***	**C908G**	**0.0018**	-	NA (T, L)	NA	7
											**T232G**	**0.01060**				
											**A528G**	**0.0008**				
											**A754G**	**0.0023**				
											**A765C**	**0.0023**				
											**A197C**	**0.024**				
											**G551T**	**0.049**				
											**A555C**	**0.0494**				
											**A556G**	**0.0494**				
											**T786C**	**0.0252**				
Foltynie T et al. [45];#; t	UK Caucasian	UPDRS	62.2	194	121	315	47	268	Dyskinesia	***BDNF***	**rs6265**	**0.001**	2.12 (1.36-3.38)	NA (L)	1-2	11
Kiferle L et al. [19]	Caucasian	UPDRS, MMSE, HY	62.69±11.52	59	63	312	60	62	Visual hallucinations/ Psychosis (psy.)	***SLC6A4***	**rs25531**	**>0.01**	**0.86 (0.52-1.44)** ^*^	(L)-259 ± 117.30 (psy.), 278.2 ± 181.98 (no psy.); (DA) 2.98 ± 1.73 (psy.), 2.78 ± 1.66 (no psy.)	≥ 4	13
										***HTR2A***	**rs6313**	**>0.05**	**0.94 (0.57- 1.55)** ^*^			
Stefanovic M et al. [29] $	Croatian	HY (2.5)	62	81	105	41 case, 145 control	NA	NA	Wearing on- off, Dyskinesia	***CYP2D6***	*3, *4, *6, *7, and *8	0.03 (*4)	**2.1 (1.11-3.99)**	NA (L)	NA	5
De Bonisa ML et al. [36]	Italian	UPDRS, HY (1.5-3)	71.96±4.69 (A1), 65.75±9.60 (A2),	38	18	44 (treated)	NA	NA	Hyperhomocysteinemia	***MTHFR***	**rs1801133**	**< 0.0001**	**-**	NA (L)	NA	10
Schuh AFS et al. [28]	Brazilian	MMSE, HY	68.0±10.3	100	96	196	50	146	Visual hallucinations	***DAT1***	**rs28363170**	**0.02**	**2.5 (1.13–5.5)**	793.2 ± 409.1 (L)	> 1	12
Fujii C et al. [30]; $; a	Japanese	NR	68.2±9.2 cases, 64.0±9.0 controls	130	81	116 case, 95 control	23	93	Hallucination	***CCK***	**rs1799923**	**0.02**	**0.28 (0.10-0.77)** ^*^	350.4 ± 140.7 (L)	3.9 ± 4.5	10
Yuan RY et al.[31]	Taiwanese	HY (1-3)	71.37 ± 9.86	85	101	76 cases, 110 control	48	28	Hyper-homocysteinemia	***MTHFR***	**rs1801133 (C677T),**	**CT-0.004 TT-0.02**	-	360.21 ± 137.62 (L, A, S/R)	6.23 ± 4.33	11
											**rs1801131 (A1298C)**	**AA <0.001 AC-0.01**	-			
Paus S et al.[38]	German	HY	64.7 ± 10.1	364	227	591	117	474	Chorea	***DRD3***	**rs6280**	**0.0005**	**-**	NA (L)	NA	13
							92	499	Dystonia							
Ivanova SA et al.[20] $	Caucasian	AIMS	NA	NA	NA	143	143	NA	Dyskinesia	***GRIN2A***				NA (L, DA)	≥ 3	7
											**rs7192557**	**0.0062**	**3.21 (1.37-7.51)**			
											**rs8057394**	**0.0033**	**3.59 (1.48-8.71)**			
De Luca V et al. [60]; t	Southern Italian	UPDRS, HY, MMSE	70.87 ± 7.59	65	66	131	47	84	Hallucination	***HOMER1***	**rs4704559**	**0.004**	**5.89 (1.33-26.14)** ^*^	676.42 ± 244.38 (L)	6 months	12
											**rs4704560**	**0.04**	**1.79 (1.03-3.10)** ^*^			
Wu H et al. [33]	Chinese	NA	NA	144	115	516	259 cases	257 control	Wearing off	***COMT***	**rs4680**	**GA vs AA-0.01**	**6.54 (1.49-28.57)**	407.45 (Multiple)	NA	10
												**GG vs AA-<0.001**	**8.84 (4.74-16.39)**			
de Lau L M et al. [37]; t	Dutch	HY, UPDRS, MMSE	49.9	143	76	219	98	121	Dyskinesia	***COMT***	**rs4680**	**A allele-0.004**	**-**	(A-P M)	NA	10
Zappia M et al. [59]	Italian	UPDRS, HY	65.2 ± 8.4	123	92	215	105	110	Dyskinesia	***DRD2***	**13, 14 + CA**_**n**_ **STR repeat**	**0.005**	**0.45 (0.26-0.79)**	654.5 ± 289.6 (L)	0.5	12
Kaplan N et al. [68]	Israeli	NR	55.2 ±13.5	213	139	352	192	160	Dyskinesia	***SLC6A3***	**rs393795**	**0.000041**	**4.96 (2.3-10.9)**	NA (L)	5 ± 4.5	11
Greenbaum L et al. [46]	Jewish Israeli, Italian	UPDRS	NR	230	160	390	128	75	Tardive dyskinesia	***ABCC8***	**rs886292**	**0.05**, 0.88	**1.63 (1–2.67)**, 1.03 (0.75–1.41)	NA (L)	≥ 3	12
										***RYR1***	**rs11880894**	0.26, **0.03**	0.7 (0.39–1.29), **1.26 (0.81–1.97)**			
										***DRD2***	**rs1800497**	0.53, **0.04**	1.25 (0.63–2.48), **0.64 (0.42–0.98)**			

*M* male, *F* female, *ADR* Adverse drug reaction, *FP* Follow-up Period, *AJ* Ashkenazi Jews; UKPDS-BBC,UK Parkinson’s disease society Brain Bank Criteria, *UPDRS* Unified Parkinson’s disease rating scale; *HY* Hoehn and Yahr Staging of Parkinson's Disease, *MMSE* Mini mental state examination, *AIMS* Abnormal Involuntary Movement Scale, *PPRS* Parkinson’s Psychosis Rating Scale; PDSK,DDSK^14^; *ADL* Activities of Daily living, *WHO-UMC* World health organization-Uppsala Monitoring Centre, *PCR-RFLP* Polymerase chain reaction- Restriction fragment length polymorphism; *OR* Odds Ratio, *CI* Confidence Interval; Drugs are L-levodopa, C-carbidopa, A-amantadine, T-, DA-Dopamine Agonist, MBI-MAO-B inhibitor, S-Selegiline, R-Ropinirole, E-Entacapone, P-Pramipexole, p- pergolide; LEDD, Levodopa equivalent drug dose; *NA* No association, -; Insufficient data. Score- Cumulative score for Methodological Quality Assessment (Ref Additional file 4: Table S1a for detailed scoring) Odds Ratio, Prevalence Ratio and Hazard Ratio are synonymously used in the table. *OR calculated using reported frequencies from the respective article. Dose of drug are in mg/day. ^a^Unit of Age, Dose and Follow up period are represented with Mean  ± standard deviation; Greenbaum L et al. two p-values are of Israeli and Italian, respectively. All the studies recruited PD patients diagnosed by United Kingdom Parkinson’s Disease society brain bank criteria expect $- Not reported, #- by Neurologist/PD Specialist, €- CAPIT, β- Gelb Criteria. Most of the studies followed PCR-RFLP for genotyping except t- TaqMan, s- Sequenom iPLEXTM, r- RT-PCR, a- ABI PRISM 310. Bold are significant polymorphisms (p≤ 0.05) and their corresponding genes

**Table 2:** Characteristics of included studies for assessment of association between genetic variants and LR in PD

Study	Population/Ethnicity	Response criteria	Age^a^ (years)	Gender		Number of samples			Genes	Studied variants	p-value	OR (95% CI)	Dose^a^ (Drug)	Treatment length (year)	Score
				M	F	Total	R	NR							
Tan EK et al. [48]	Singapore	UPDRS	69.9±7.6	24	15	39	NA	NA	***COMT***	**rs4680**	**0.004**	-	7.37 mg/ week (P); 421.5 ± 226.2mg (L)	at least 3 months	12
Liu YZ et al. [51]	Chinese	UPDRS-I, HY <2.5	61.90 ± 8.20	14	16	30	11	19	***DRD3***	**rs6280 (Ser/Ser)**	**0.024**	**9.75( 1.60- 59.70)** ^*^	0.125mg/thrice a day (L, B,P)	> 3 months	12
Devos D et al. [52]	Caucasian	UPDRS-III, HY	>30	23	10	33	14	19	***DDC***	**rs921451**	**0.048**	-	NA (L,B)	NA	13
							8	25		**rs3837091**					
Moreau C et al. [54]	French	UPDRS II, III	60-63	NA	NA	61	NA	NA	***SLC6A3***	**rs28363170**	**0.005**	-	710 ± 90.8mg/day (L)	16-17	12
										**rs3836790**	**<0.001**	-			

*M* male, *F* female, *R* responder, *NR* non-responder, *bp* base pair; Dose of drug are in mg/day. Unit of Age, Dose and Follow up period are represented with Mean  ± standard deviation; ^a^ unit of age and dose are represented with Mean ± standard deviation﻿; OR, Odds Ratio; *OR calculated using reported frequencies from the respective article; *UPDRS* Unified Parkinson’s disease rating scale, *HY* Hoehn and Yahr Staging of Parkinson's Disease; Drugs are L-levodopa, C-carbidopa, A-amantadine, T-, DA-Dopamine Agonist, MBI-MAO-B inhibitor, S-Selegiline, R-Ropinirole, E-Entacapone, P-Pramipexole; PCR-RFLP, Polymerase chain reaction- Restriction fragment length polymorphism. *NA* No Association; -,Insufficient data. Bold are significant polymorphisms (p≤ 0.05) and their corresponding genes

On a systematic analysis of the GWAS, conducted so far on PD susceptibility risk, included twenty GWAS studies (consisting of 45,465 cases and 173,222 controls). Sixty-one loci have been identified as significantly associated with the disease risk (*p* ≤ 0.01 × 10^−8^) (Table 3). A detailed summary of these significant genetic variants obtained from GWAS in the field of PD has been represented in Additional file 4: Table S1. The genes *BST1*, *CCDC62*/*HIP1R*, *DGKQ*/*GAK*, *GBA*, *ITGA8*, *LRRK2*, *MAPT*, *MCCC1*/*LAMP3*, *PARK16*, *SNCA*, *STK39*, and *SYT11*/*RAB25* are disease risk loci following the collaborative meta-analyses. *SNCA* (*p* ≤ 4.16 × 10^−73^) and *MAPT* (*p* ≤ 2.37 × 10^−48^) have been studied in 9 and 7 GWA studies establishing the functional relevance in the disease physiology, making them the most prominent loci. Followed by *LRRK2* and *GAK* in 4 studies, *GBA/ SYT11* and *MCCC1/ LAMP3* in 3 studies respectively.

**Table 3:** Significant markers from GWAS studies on Parkinson's disease susceptibility risk

Study	Gender		Age	Discovery				Follow-up			# Genes	Genes	Variants	p-value	OR
	M	F		# SNPs	Population	# Cases	# Ctrls	Population	# Cases	# Ctrls					
Edwards TL et al. [85]	381	224	64.56 ± 12.18	4,22,322	Caucasian-MIHG	605	621				16	*PLEKHM1*	rs11012	5.65×10^−8^	0.70
												*SNCA*	rs2736990	6.74×10^−8^	1.29
Satake W et al. [86]				4,35,470	Japanese	1,078	2,628	Japanese	612	14,139	4	*PARK16*	rs823128	4.88×10^−9^	1.41
													rs823122	5.22×10^−8^	1.37
													rs947211	1.52×10^−12^	1.30
													rs823156	3.60×10^−9^	1.37
													rs708730	2.43×10^−8^	1.33
												4p15	rs11931532	5.13×10^−9^	1.24
												*BST1*	rs12645693	8.65×10^−9^	1.24
													rs4698412	1.78×10^−8^	1.24
													rs4538475	3.94×10^−9^	1.24
												4q22	rs11931074	7.35×10^−17^	1.37
								Japanese	321	1,614		*SNCA*	rs3857059	5.68×10^−16^	1.36
													rs6532194	4.15×10^−13^	1.32
												12q12	rs1994090	2.72×10^−8^	1.39
												*LRRK2*	rs7304279	5.06×10^−8^	1.38
													rs2708453	9.67×10^−8^	1.38
													rs2046932	4.34×10^−8^	1.39
Sanchez JS et al. [87]	515.27	472.73	55.9±15.1	4,63,185	stage 1 USA	988	3071				3	*MAPT*	rs393152	1.95×10^−16^	0.77
													rs199533	1.09×10^−14^	0.78
													rs17563986	1.67×10^−14^	0.78
	452.98	304.02	56±11.64		Germany	757	976					*SNCA*	rs2736990	2.24×10^−14^	1.23
													rs3857059	3.74×10^−15^	1.48
													rs11931074	1.62×10^−14^	1.46
	1083.8	444.19	62.5±8.55		stage 2 USA	1528	2044					OTHERS	rs823128	7.29×10^−8^	0.66
Do CB et al. [88]	2065.9	1360.122	64.3±10.6	5,22,782	primarily European (23andMe)	3426	29624	European descent (IPDGC)	6584	15470	11	*LRRK2*	rs34637584	1.82x10^-28^	9.615
												*GBA*	i4000416	5.17x10^-21^	4.048
												*SNCA*	rs356220	2.29x10^-19^	1.285
												*MAPT*	rs12185268	2.72x10^-14^	0.769
												*MCCC1*/ *LAMP3*	rs10513789	2.67x10^-10^	0.803
												*SCARB2*	rs6812193	7.55x10^-10^	0.839
												*GAK*	rs6599389	3.87x10^-8^	1.311
												*SREBF1*/*RAI1*	rs11868035	5.61x10^-8^	0.851
Burns EMH et al. [89]	1063	502	67.59±10.68	7.2 million	USA (NGRC cohort)	1565	1986	White, non-Hispanics (NINDS cohort)	621,102	797	4	*SNCA*	rs356220	1.00x10^-9^	1.37
												*HLA*	rs3129882	5.00×10^-10^	1.38
IPDGC [90]	577.75	393.255	55.9±15.1	76,89,524	USA-NIA	971	3034	US	2,807	2,215	11	*SYT11*	chr1:154105678	3.50×10^-12^	1.47
	966.74	738.265	65.8±10.8		UK	1705	5200	UK	1,271	1,864		*AMCSD*	rs6710823	6.75×10^-9^	1.1
	446.68	295.316	56±11.6		Germany	742	944	Dutch	304	402		*STK39*	rs2102808	4.23×10^-10^	1.18
	610.93	428.068	48.9±12.8		France	1039	1984	German	1153	712		*MCCC1/ LAMP3*	rs11711441	8.04×10^-12^	0.84
	522.1	353.904	61.5±9.2		USA- dbGAP	876	857	French	267	363		*GAK*	chr4:911311	3.67×10^-12^	1.16
												*BST1*	rs11724635	1.21×10^-16^	0.87
												***SNCA***	**rs356219**	**1.82×10** ^**-47**^	1.29
												*HLA-DR*	chr6:32588205	2.24×10^-14^	0.78
												*LRRK2*	rs1491942	6.01×10^-14^	1.27
												*CCDC62/ HIP1R*	rs12817488	3.20×10^-13^	1.14
												*MAPT*	rs2942168	1.47×10^-28^	0.79
Nalls MA et al. [69]	315	289	62.2±12.3	78,93,274	IPDGC-DC	604	4,916	IPDGC-FR	553	474	32	*GBA/SYT1*	rs35749011	1.37×10^-29^	1.824
	579.18	405.82	48.9±12.8		IPDGC-FR	985	1984	IPDGC-GE	1044	871		*RAB7L1/ NUCKS1*	rs823118	1.66×10^-16^	1.122
	401.53	265.466	55.7±11.5		IPDGC-GE	667	937	IPDGC-GK	944	877		*SIPA1L2*	rs10797576	4.87×10^-10^	1.131
	476.16	267.84	55.6 ± 11.8		IPDGC-NE	744	2019	IPDGC-NIA	2407	2782		*ACMSD/ TMEM163*	rs6430538	9.13×10^-20^	0.875
	557.52	379.485	57.8±13.2		IPDGC-NIA	937	1896					*STK39*	rs1474055	1.15×10^-20^	1.214
	1972.9	1288.095	64.2±11.2		23andMe.v2	3261	29499	IPDGC-UK	405	547		*MCCC1*	rs12637471	2.14×10^-21^	0.842
	528.26	337.74	63.9±10.9		23andMe.v3	866	32538					***TMEM175/ GAK/ DGKQ***	**rs34311866**	**1.02×10** ^**-43**^	0.786
	179.02	88.976	59.9±12.1		Ash Jewish	268	178					*BST1*	rs11724635	9.44×10^-18^	1.126
	362.19	211.806	57.2±12.03		HIHG	574	619					*FAM47E/SCARB2*	rs6812193	2.95×10^-11^	0.907
	1306.6	649.392	58.6±11.7		NGRC	1956	1982					***SNCA***	**rs356182**	**4.16×10** ^**-73**^	0.76
	495.97	332.028	62.1±10.7		PGPD	828	852					*HLA-DQB1*	rs9275326	1.19×10^-12^	0.826
	58.957	48.043	73.0 ± 5.1		CHARGE-CHS	107	3164					*GPNMB*	rs199347	1.18×10^-12^	1.11
	34.98	25.02	76.2 ± 10.8		CHARGE-FHS	60	3889					*INPP5F*	rs117896735	4.34×10^-13^	1.624
												*MIR4697*	rs329648	9.83×10^-12^	1.105
												*LRRK2*	rs76904798	5.24×10^-14^	1.155
												*CCDC62*	rs11060180	6.02×10^-12^	1.105
												*GCH1*	rs11158026	5.85×10^-11^	0.904
												*TMEM229B*	rs1555399	6.63×10^-14^	0.897
												*VPS13C*	rs2414739	1.23×10^-11^	1.113
												*BCKDK/ STX1B*	rs14235	2.43×10^-12^	1.103
												***MAPT***	**rs17649553**	**2.37×10** ^**-48**^	0.769
												*RIT2*	rs12456492	7.74×10^-12^	0.904
												*DDRGK1*	rs8118008	3.04×10^-11^	1.111
												*FGF20*	rs591323	6.68×10^-8^	0.916
Pankratz N et al. [91]	NA	NA	NA	2633913		4,238	4,239		3,738	2,111	6	*GBA*	E326K	5.00×10^−8^	1.71
												*GAK*	rs11248060	3.00×10^−9^	1.26
												***SNCA***	**rs356220**	**8.00× 10** ^**−35**^	1.38
													rs356198	5.00×10^−9^	0.82
												*HLA reg*	rs2395163	3.00×10^−11^	0.81
												*MAPT*	rs199515	3.00×10^−17^	0.76
												*RIT2*	rs12456492	2.00×10^−10^	1.19
Hamza TH et al. [92]	1346	654	58.34±11.93	8,11,597	Caucasian	2000	1986	NA	NA	NA	6	*HLA-DRA*	rs3129882	1.90×10^−10^	1.26
												*GAK9*	rs11248051	3.20×10^−9^	1.46
												*SNCA*	rs356220	3.40×10^−11^	1.38
Spencer CCA et al. [93]	1244.2	460.81	65.8	532 588	UK	1705	5175	French	1039	1984	6	*SNCA*	rs2736990	1.36×10^-27^	1.23
												*MAPT*	rs393152	4.75×10^-28^	1.31
Vacic V et al. [94]				haplotypes	Ashkenazi jews	1,130	1,807	Ashkenazi Jews	306	2583	7	*MAPT*	rs17577094	4.51×10^-10^	0.64

#, Number of; (D),Discovery; Ctrls, control; MIHG, Miami Institute for Human Genomics; IPDGC, International Parkinson's Disease Genomics Consortium; NGRC, NeuroGenetics Research consortium; NIA, National Institute on Aging; dbGAP, database of Genotypes and Phenotypes; HIHG, Hussman Institute of Human Genomics; PGPD, Physician Group Practice Demonstration; CHARGE-CHS, Cohorts for Health and Aging Research in Genetic Epidemiology; NA, Not applicable;

Two reviewers (DG and MKM) independently assessed the methodological quality of all the selected articles using a predefined set of criteria. All included studies were assessed for the quality of data presented by using modified criteria suggested by Wells K. et al. [24]. The quality assessment was scored on 14 parameters (Additional file 1: s5 and s6), with a positive score awarded for each detail present in study, the lack of detail was described as either NA (not applicable) or NR (not reported). NA was assessed with an equal positive score and was given only when the study was deemed independent of the parameter; NR was equated to no scoring and was independently awarded by the authors if the methodology was found insufficient or unreported. The detailed list of the 14 parameters used for the quality assessment has been discussed in Additional file 1: s7. Conflicting scores were reached to a consensus upon discussing (RK and PT). If the score was obtained as 11 or higher, the study was ranked as high quality.

### Protein-protein interaction network

To decipher the connecting molecular link between the roles of the genes studied for LR and disease risk would help us rule out the bias if any to identify the interacting proteins of drug response thus elucidate the genetic landscape of the disease. Two independent protein-protein interaction (PPI) networks were constructed using STRING application with minimum required interaction score of 0.7 (high confidence), active interaction sources were set for only known interactions (databases and experiments) and maximum number of interactors to show in the 1st shell- no more than 50, and no 2nd shell interaction [25]. Pathway enrichment analysis was conducted by WebGestalt [26]. Using pathway commons enrichment analysis, default GO slim classification, 0.001 significance level, and minimum no. of genes in a category was set at 5. The enrichment analysis was run adjusting the false discovery rate (FDR) using the Benjamini-Hochberg (BH) procedure to obtain the results independently for the two set of genes.

## Results

### Search and study selection

**Fig. 1:**
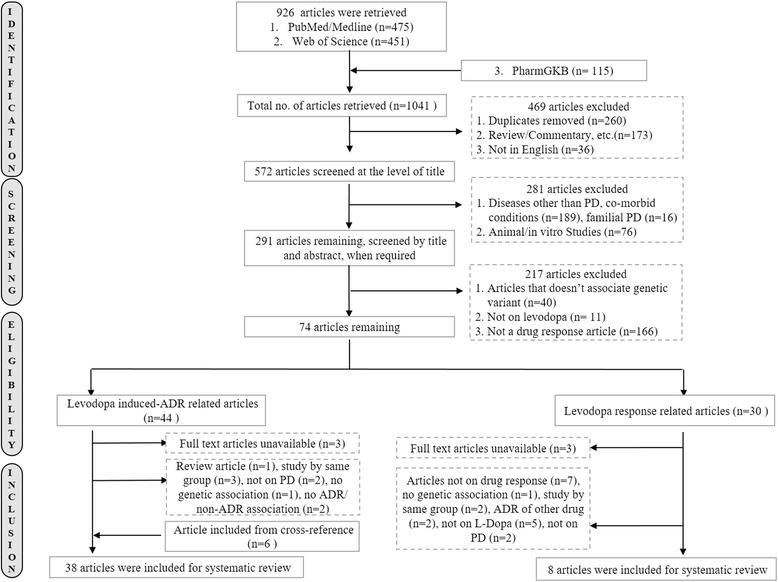
Flow diagram representing the selection of studies for systematic review of levodopa response studies

The workflow of the search and study selection has been represented in Fig. 1. A total of 1041 articles were obtained, of which 469 studies were excluded that includes duplicates, reviews, and articles in other languages (*n* = 469). The remaining 572 articles were screened by title and abstract following the inclusion and exclusion criteria resulting in further exclusion of 498 articles: 189 studies discussing other diseases or comorbid conditions, 16 studies on familial PD were removed as current study focuses on sporadic form of the disease, 76 studies were based on animal or in vitro models for PD, 40 studies were not on genetic association and 166 others did not discuss any drug response, finally 11 papers on drugs other than levodopa were also excluded to narrow down the scope of current study to the most widely prescribed medication. A total of 74 eligible publications were further divided into studies that are on adverse effect of levodopa or the efficacy of the drug in the patient cohort. In case of unavailable full text articles, the authors were contacted (*n* = 22). Responses received (*n* = 16) were included in the study, rest excluded (*n* = 6). From the cross-references of the included studies, six additional articles fulfilled the inclusion criteria [19, 27–31]. Thus, finally 38 eligible publications on levodopa induced ADR and 8 on drug response had sufficient data available for extraction to carry forward the systematic review.

### Study characteristics

The methodological and demographic characteristics of the ADR studies and the drug efficacy related studies of levodopa are summarised in Table 1 and Table 2 respectively [complete in Additional file 2: Table S1(8a) and Additional file 3: Table S2(8b)]. A total of 4127 subjects with ADRs were enrolled in the 37 studies. Most of the studies included were cohort studies except four which were case-control studies [29, 32–34]. Apart from original research articles, four letters [19, 21, 35, 36], two brief reports [37, 38], and two short communications [33, 39] were included as they had sufficient data pertaining to our criteria of inclusion and exclusion, one randomized control trial [40] was included. All the patients recruited in the independent studies were primarily administered with levodopa alone (*n* = 21), dopamine agonist (*n* = 4), DDC inhibitor/ carbidopa (*n* = 4), COMT inhibitor/ entacapone/tolcapone (*n* = 5) or MAO-B inhibitor (*n* = 1). The dose of levodopa administered ranged between 200.00 to 805.14 mg/day. The range of follow up period for the recruited subjects in the studies was between 6 to 10.3 months. The PD subjects recruited were diagnosed by UK Brain Bank Criteria (UK BBC) [41] in 26 articles, by Gelb’s criteria [42] in 1 article, by CAPIT [43] (Core assessment programme for intra-cerebral transplantations) in 1 article or by an experienced neurologist in 2 articles. The different motor functioning assessment scales used are provided in Additional file 2: Table S1(8a). The variability in HY assessment scale of 3.5 or more have been used.

Out of thirty-seven studies, thirteen focussed on levodopa induced dyskinesia exclusively, three on other motor fluctuation exclusively, one on wearing on/off, three on hyper-homocysteinemia, five studies were on hallucination, one study each on COMT inhibitor induced toxicity and elevated liver transaminase levels, and twelve studies discussed multiple ADR in the same cohort of recruited patients. Motor fluctuations were observed to be the most common adverse effect of anti-Parkinson’s medications. Dyskinesia (subjects of tardive dyskinesia, peak dose dyskinesia, diaphasic dyskinesia were grouped together) being the most prevalent among the subjects with ADRs, was present in 45.72% of patients. This group had an early age at onset of motor symptoms, longer disease duration and 560.96 ± 321.97 mg/day levodopa daily mean doses. In addition to levodopa, around one-third of the total patients were administered with COMT or MAO-B inhibitor or DDC inhibitor like carbidopa, entacapone or tolcapone. In addition to dyskinesia, adverse effects like other motor complications (motor impulsivity, wearing on-off, chorea, dystonia) were observed in 35.74% of total ADR subjects, and hyper-homocysteinemia 2.62%. Hallucinations occur as a consequence of psychosis, hence the two have been synonymously used and their subjects have been summed up together constituting 12.38%. One paper each discussed about COMT inhibitor induced toxicity (0.26%) and elevated transaminase level (3.27%).

Europeans (EUR) constituted as the major population of the studied subjects comprising 47.47%, followed by East Asian (EAS) at 26.52%, South Asian (SAS) at 13.32% closely followed by American (AMR) 12.66%. Most of the studies recruited subjects of the same population except Cheshire P. et al. (2013) [44] included patients from UK and Australia, Foltynie T. et al. (2014) [45] included all UK Caucasians, one Afro-Carribean, two Asian-Indian and one half Caucasian and half Asian-Indian, Greenbaum L. et al. (2013) [46] included Jewish and Italian subjects and Ziegler DA. et al. (2014) [47] included 122 American and 1 Asian.

A total of 645 subjects have been include in the 8 studies of levodopa efficacy. Of these, three studies are brief communications [48–50]; three were cohort studies [51–53] and one each of RCT [54], and letter [55]. Three studies recruited patients administered with levodopa alone [49, 53, 54], four studies prescribed levodopa with DDC inhibitors like Benserazide [50–52], Pyridoxine [48], and one on Dopamine agonist (Pramipexole) [51] and COMT inhibitor (carbidopa) [55]. The mean levodopa dose administered is 356.8 mg/day. All the patients were diagnosed by UK BBC [41], expect Moreau C. et al. [54] by Gibb’s criteria [56], Tan EK et al. [48] and Xie T et al. [49] by neurologists and Devos D. et al. [52] do not mention the diagnosing criteria. Two hundred twenty seven subjects responded to the therapy assessed by the scoring criteria. The study populations included Asians (54.57%) and Europeans (45.42%).

### Methodological quality

The cumulative quality assessment score obtained by individual ADR studies are represented in Additional file 2: Table S1(8a) and that of drug efficacy in Additional file 3: Table S2(8b). In ADR studies, the mean methodological assessment score was calculated to be 10.56 (SD 2.15), range 7 to 13. On the modified scale, twenty of thirty seven articles were deemed as good quality with a cut off score of ≥11, thirteen articles scoring ≥9–10 were categorised under moderate quality and finally any scores below 9 were judged as poor quality which included four articles. In L-dopa efficacy studies, the mean methodological score was 11.13 (SD 1.86), ranging 7 to 13. Six articles qualified to be good, and one each as moderate and poor quality.

### Genetic factors in levodopa induced adverse effects

From total publications, 40 variants within 18 genes (*HOMER1, ADORA2A, ANKK1, MTHFR, DRD2, SLC6A3, COMT, UGT1A, ACE2, BDNF, ABCC8, RYR1, DRD3, GRIN2A, SLC6A4, HTR2A, CYP2D6, CCK*) were found to have significant association (*p* ≤ 0.05) with any type of levodopa induced ADR in PD (Additional file 2: Table S1(8a)).

Europeans (EUR) studies included 4258 subjects and reported 30 variants associated with ADRs. Among EUR, 1347 subjects were Italian from 8 studies [32, 35, 36, 46, 57–60]. With 13, 14 CA_n_ STR repeats in *DRD2* gene being the most significant variant associated with dyskinesia, followed by rs1801133 (*MTHFR*) associated with hyper-homocysteinemia, rs474559 (*HOMER1*) with hallucinations. Other polymorphisms including rs886292 (*ABCC8*), rs11880894 (*RYR1*), rs1800497 (*DRD2*), rs6265 (*BDNF*), rs11646587, rs7192557 and rs8057394 (*GRIN2A*) were found to be associated with dyskinesia in patients administered with levodopa medication. 5-HTTLPR and rs6313 (*5-HTR2A*) were reported in higher frequency in patients with psychosis and Acuña G. et al. (2002) [61] suggested the association of 10 *UGT1A* SNPs with elevated liver transaminase level. Among East Asians (EAS), two Chinese studies [62, 63] constituting 746 subjects concluded, rs4680 (*COMT*) and I/D polymorphism (*ACE2*) associated in patients with wearing on-off and psychosis respectively. A Japanese study [30] showed significant association with -45C/T (*CCK*) in patients with hallucination and rs1801131, rs1801133 (*MTHFR*) with elevated plasma homocysteine levels in a Taiwanese study [31]. In American (AMR) population, Brazilians constituted four studies [28, 64–66] determining rs4704559 (*HOMER1*), rs2298383 (*ADORA2A*), rs1800497 (*ANKK1*) associated with dyskinesia, rs3761422 (*ADORA2A*) with motor fluctuation and rs28363170 (*DAT1*) with hallucination. Among South Asian (SAS) population, Israelis were most abundantly studied representing association of rs393795 (*DAT1*), rs886292 (*ABCC8*), rs11880894 (*RYR1*) and rs1800497 (*DRD2*) with dyskinesia [39, 46, 67, 68].

### Genetic factors in other LR

On elaborate systematic extraction of published literature on LR, in terms of efficacy, of the drug, only eight studies deemed our defined inclusion and exclusion criteria. The enzymes directly involved in the metabolism and activity of levodopa is evidently been mostly studied with the altered LR. rs4680 (*COMT*) [48], rs6280 (*DRD3*) [51], rs921451, rs3837091 (*DDC*) [52], rs28363170, rs3836790 (*SLC6A3*) [54] were the significant variants with reduced LR (Additional file 3: Table S2(8b)). However, no conclusive results could be drawn from this systematic analysis due to large variability and low significance.

### Genetics of PD susceptibility

Employing GWAS dataset to stratify disease susceptibility loci, we follow an unbiased approach to identify such loci in sporadic PD cases. Nalls et al. (2014) [69] recently conducted a large scale meta-analysis to identify the associated loci with disease risk. Keeping this study as the base of the systematic review of all the GWAS on PD risk, and adding the recent studies to it. Twenty studies were included for the systematic review with 45,465 cases and 173,222 controls, mostly from including Caucasian population followed by Jewish, Chinese and Japanese. Sixty one loci in genes like *BST1*, *CCDC62*/*HIP1R*, *TMEM175/DGKQ*/*GAK*, *GBA*, *ITGA8*, *LRRK2*, *MAPT*, *MCCC1*/*LAMP3*, *PARK16*, *SNCA*, *STK39*, and *SYT11*/*RAB25* were associated with the disease susceptibility as shown in Table 3. Additional file 4: Table S1 tabulates all the loci found to be associated with disease susceptibility and the significant single nucleotide polymorphism (SNPs) in bold (*p* value ≤1.0 × 10^−8^).

### Protein-protein interaction network

**Fig. 2:**
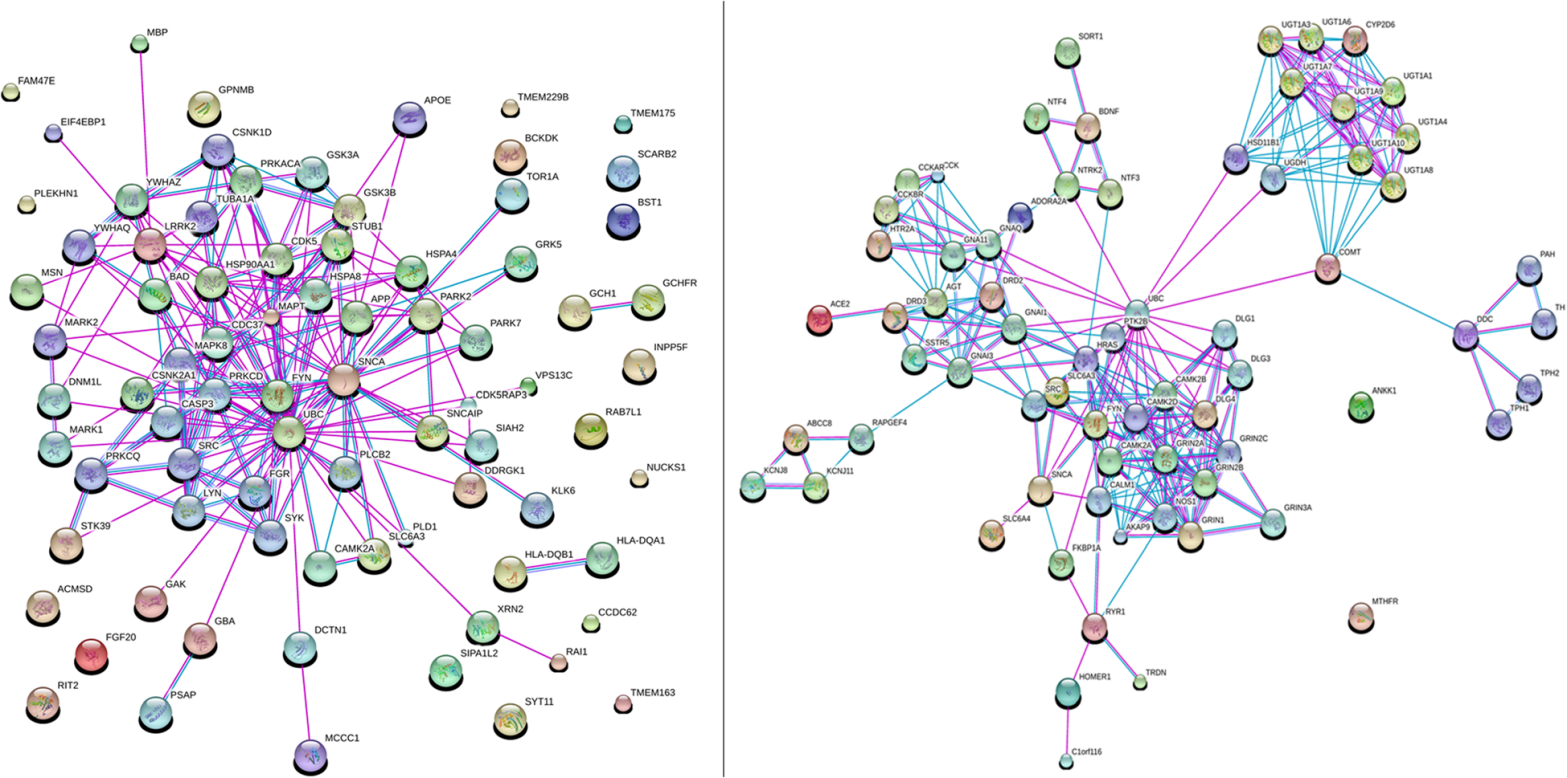
Protein-protein interaction network between PD response related genes and disease related genes, respectively. Networks representing protein-protein interaction of gene modules identified from (a) Parkinson’s disease susceptibility genes from GWAS studies and (b) levodopa response genes identified from systematic review. The lines with different colours represent data annotated based on different association evidence. The colour coding are Query proteins- red, other coloured nodes are 1st shell interactors. Interacting lines- blue- from curated databases, pink- experimentally determined

**Fig. 3:**
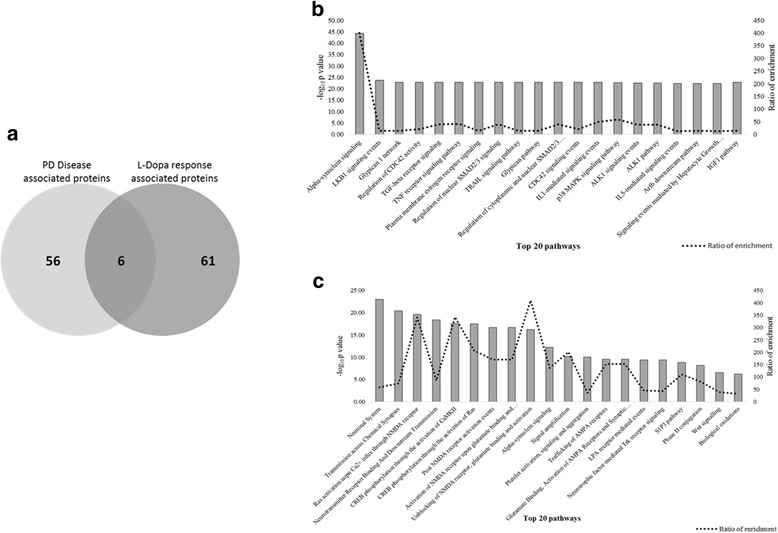
Enrichment analysis of disease associated and L-Dopa response related proteins. (a) Represents the overlap between the two protein sets. Plot 2(b) & (c) represents the top 20 pathways with PD and L-Dopa response, respectively

We performed PPI analysis using genes obtained from the systematic review of LR and disease risk in order to understand the functional association among the genes in the respective gene modules. With 19 proteins associated with LR and 35 with PD, two independent PPI networks were constructed respectively using STRING database to identify critical candidate genes/proteins (Fig. 2). In LR, the 67 nodes, represent the genes, and 263 edges weight the likelihood of nodes in common biological functions.) In PD susceptibility, 62 nodes linked with 190 edges are depicted. Functional enrichment analysis using WebGestalt identified three common pathways (Alpha synuclein signalling, ADP-ribosylation factor 6 (*Arf6*) downstream pathway, and Insulin-like growth factor 1 (IGF1) pathway) in the top 20 pathways for LR and PD (Fig. 3). The common six proteins, *UBC*, *SNCA*, *FYN*, *SRC*, *SLC6A3*, *CAMK2A*, between LR and PD were thus obtained. The functional significance of the identified nodes in PPIs are clearly substantiated by the biological processes obtained from functional enrichment analysis.

## Discussion

PD is a progressive brain disease which causes significant movement disability [70]. The treatment is aimed at symptomatic management rather than complete cure. However, the challenge is large clinical variability in drug response and adverse effects on prolonged therapy. Discerning the genetic factors responsible for this variability to the drug toxicity and efficacy can provide better clinical management. This study identifies such genetic variants in genes involved in L-dopa metabolism and in the disease etiology by a systematic review approach. Further from the limited number of genes obtained from the systematic review, we extended our effort to integrated computational approaches like network modelling and functional enrichment to identify the other interacting proteins and thereby distinguish the common proteins and molecular pathways that participate in LR and the disease. We additionally show the limitations of the published literature and give insights that may be useful to future studies.

We have implemented a modified scale of Wells K et al. (2009) [24] criteria with five additional parameters, to assess the quality of articles included in the systematic review. To the best of our knowledge our study has incorporated the most comprehensive methodological quality assessment scoring for screening articles of systematic review. Candidate gene studies have been screened for the systematic review of LR.

**Fig. 4:**
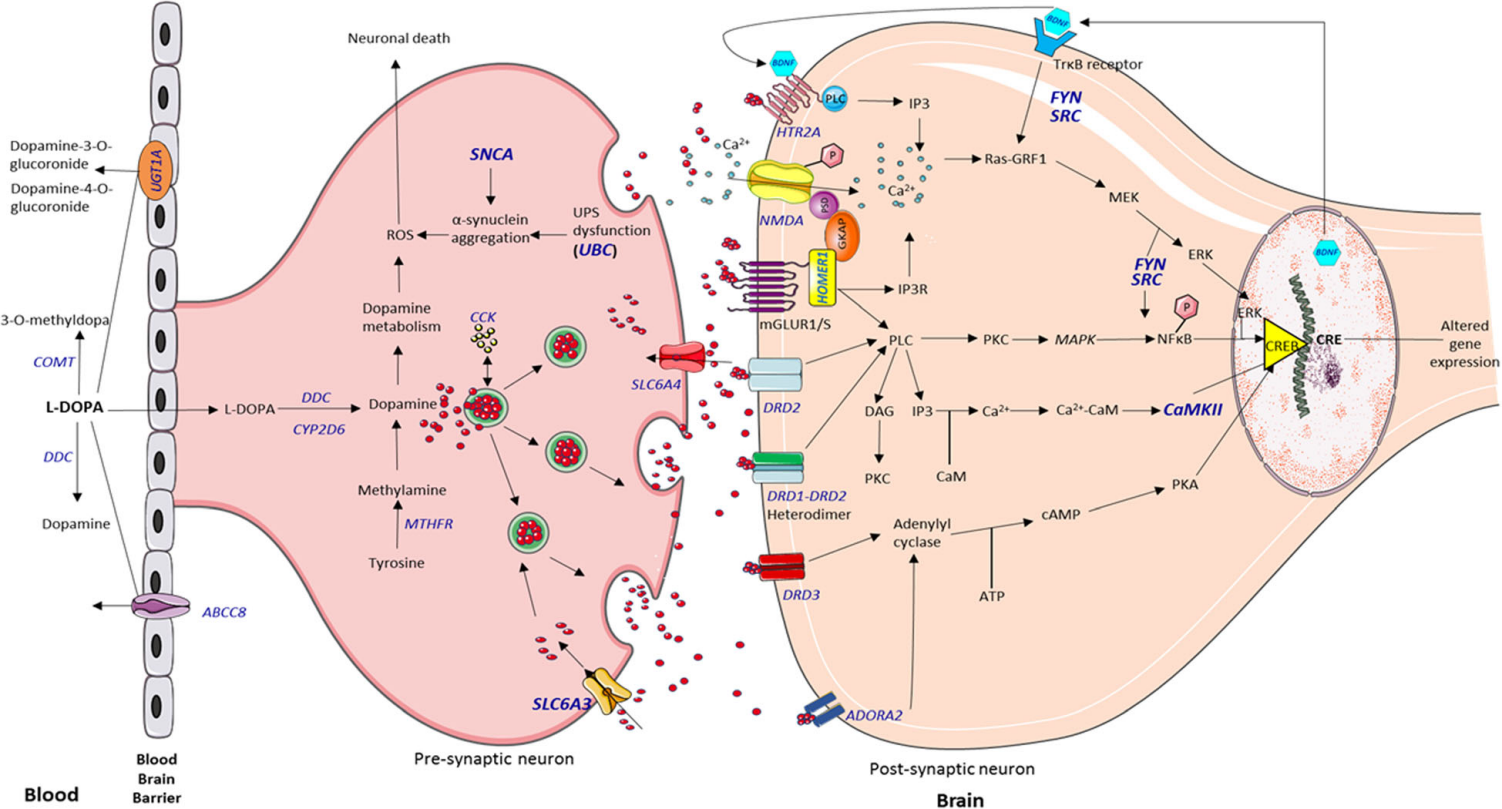
Crosstalk between the genes (in blue) obtained from the systematic review of LR and their biological functions in the dopaminergic neurons. Levodopa is metabolized by *COMT* and *DDC.* Dopamine in the pre-synaptic neuron is produced from and released into the synapse by exocytosis. The receptors in the post-synaptic neurons [*DRD1, DRD2, DRD3, ADORA2,* glutamate receptors (*mGLUR1/S*)*, NMDA, HTR2A*] uptake dopamine and further downstream cellular signalling leads to altered gene expression. Signalling pathways like the Ras-GRF1 mediated signalling, Ca^2+^-calmodulin dependent pathway and adenylyl cyclase participate in dopaminergic response. The function of 6 common genes (in bold) found with overlapping roles in LR and PD risk are discussed in text

A total of 18 genes from the 37 ADR studies and 4 genes from the 8 efficacy studies were retrieved after the systematic review. Most of the genes are related to dopaminergic pathway and their role have been depicted in Fig. 4. Most of the studies included the genes related to dopaminergic pathway. For instance, among ADR studies, CA_n_ STR 13, 14 (*DRD2*) was found to be most significantly associated with dyskinesia, rs1801133 (*MTHFR*) with hyper-homocysteinemia, and rs474559 (*HOMER1*) with hallucination. Carriers of 13, 14 alleles are found to have lower risk of developing dyskinesia but role of this repeat is still unknown. Patients with the TT677 (rs1801133, 677C > T) genotype exhibit 50% reduced activity of *MTHFR* enzyme, consequently elevating the plasma homocysteine levels [32]. rs474559 G allele (*HOMER1*) have lower prevalence of dyskinesia as it might disrupt the glutamatergic transmission [60]. In efficacy related studies, rs28363170, rs3836790 (*SLC6A3*) and rs4680 (*COMT*), were important. Individuals with rs3836790 6/6 or rs28363170 10/10 (*SLC6A3*) genotypes have higher transporter expression leading to lower dopamine levels at the synapse [71]. The haplotype structure formed by four SNPs (rs6269: A > G, rs4633: C > T, rs4818: C > G, rs4680:A > G) characterises the COMT enzyme activity to low (ACCG), medium (ATCA) and high (GCGG) [72, 73]. Accordingly, the levodopa metabolism is affected, altering the synaptic dopamine concentration. Also we observed that *SLC6A3*, *COMT*, and *DRD3* genes were common between the ADR and efficacy studies resulting in 19 exclusive genes from LR studies.

In addition, to identify the disease genes involved in drug response and vice versa, genes found implicated in PD susceptibility from GWAS were also retrieved. This led to obtaining 61 significantly associated SNPs (*p* value ≤1.0 × 10^−8^) pertaining to 35 genes (Additional file 4: Table S1). Then an integrated network analysis resulted in six common molecular targets (*SNCA*, *FYN*, *SRC*, *UBC*, *CAMK2A*, and *SLC6A3*) from the overlap between 67 nodes (and 263 edges) in LR and 62 nodes (and 190 edges) in PD pathophysiology, respectively. Among the six common molecular targets, *SNCA* has been widely established to be a major player in PD susceptibility as it a major component of Lewy bodies and mutant *SNCA* has a greater tendency to acquire misfolding [70, 71]. Aggregation of *SNCA* has been shown to be neurotoxic for the cell through the formation of intermediate aggregates called protofibrils [74]. In a recent report the distinct role of alpha-synuclein forming fibrils as the major toxic, resulting in progressive motor impairment and cell death leading to neurotoxic phenotypes in PD is demonstrated [74]. *FYN*, a tyrosine kinase family protein, found inside nerve cells and helps in communicating signals or chemical instructions between different cellular components. This protein has been observed to get modified on levodopa administration, causing dyskinesia [75]. Wang et al. (2016) validated neuro-inflammation inhibition by *SRC* (SRC proto-oncogene, non-receptor tyrosine kinase) signalling pathway to be a potential drug and disease candidate which supports our finding *SRC* as the common molecular bridge to both drug response and disease pathology [76]. *UBC* belongs to the Ubiquitin family C which carries out the ubiquitin mediated proteolysis and aggregate ubiquitin monomers in the diseased brain. The ubiquitin proteins cause aberrations in the ubiquitin proteasome system (UPS) leading to PD pathogenesis [77]. The neuronal protein, CAMK2A alters with intracellular calcium ion concentration change that is abnormally activated following dopamine depletion thus modulating the neuronal function in striatum [78]. Zhang et al. (2014) also established an interaction between CaMK2A and Dopamine D2 receptors in striatal neurons, sensitive to long-term levodopa administration to PD rats [79]. Finally, the *SLC6A3/DAT1* variants have a significant effect on striatal activation and performance in PD as suggested by Habak et al. These results have furnished evidences on the role of these candidates in both levodopa metabolism impairment and disease risk. Further, these plausible biomarkers might bridge the path between levodopa metabolism and disease pathology resulting in reduced ADRs, optimum efficacy and, accurate diagnosis.

Functional enrichment analysis revealed prominently [74], alpha-synuclein pathway to be the most significant candidate with the set of disease and response related genes respectively followed by other growth factor signalling pathways like *Afr6* downstream pathway, *IGF1* pathway, and so on. *Arf6*, ADP-ribosylation factor, signalling plays a role in the Ras- mediated cell signalling [80]. It is also responsible in the intracellular trafficking of *DRD2* by GRK and PKC proteins [81]. The potential role of *IGF1*, Insulin-like growth factor 1, signalling has been studied with neurodegeneration in human, participating in functions like brain neuron survival, synaptic transmission as well as plasticity [82]. Bernhard FP. et al. (2016) [83] also established that *IGF1* might serve as a PD prediction marker, observing elevated levels of *IGF1* in PD patients. Additional file 5: s2 and Additional file 6: s3 tabulate the enriched functions obtained by levodopa response genes and PD related genes. We highlighted the potential usefulness of these biological functions in PD treatment which can be affirmed by in vitro and/or in vivo model systems.

Although significant findings have been observed in our study, several limitations exist. The papers included in the systematic review presented high heterogeneity in terms of diagnosis, response criteria, drugs administered with different doses and genotyping techniques. As suggested by Schumacher-Schuh et al. (2014) [84] the phenotypic heterogeneity in terms of adverse effects lacks clinical instrument to adequately measure the ADR, whereas in terms of efficacy, several response rating scales have been incorporated. In GWAS studies, the assayed SNPs are usually to mark a genome region that influences the studied phenotype. However, we have picked up the annotated genes corresponding to the significantly associated SNPs from the respective studies, to identify the proteins that play a role in the biological processes which ultimately influences the phenotype. Motor fluctuation, a common ADR of levodopa, lacks clear clinical classification and hence assessment. A regular record of patient motor state could be preferred. Genetic heterogeneity is another source of variability between studies because different markers in the same genes were employed for these associations; moreover, patients with different genetic backgrounds may not be strictly comparable. One major limitation of network biology is the quality and the coverage of the interactions. The rate of discovery of false positives and false negatives are high which shows the need to rank the reported interactions for further validation.

## Conclusion

In summary, the present study provides a framework for better understanding of the molecular interplay between L-Dopa metabolism with PD pathophysiology and also a means to evaluate putative biomarkers to bridge the gap in treatment outcome and disease risk. We propose the above six genes could be useful in predicting both the LR and disease risk, simultaneously. This however warrants further experimental validations to develop into a targeted therapy. Translating these evidences into future validation would present pre-diagnostic marker development which can be applicable in clinical manifestation. A definitive role of these molecular targets in the disease progression can also lead to substantive advancement in PD treatment.

## Additional files

Additional file 1: Including Supplementary Material such as Supplementary text S4, S7, Supplementary Tables S5–S6. (DOCX 34 kb)

Additional file 2: Table S1. Characteristics of included studies for assessment of association between genetic variants and ADRs in PD (DOCX 123 kb)

Additional file 3: Table S2. Characteristics of included studies for assessment of association between genetic variants and LR in PD (DOCX 52 kb)

Additional file 4: GWAS studies on Parkinson’s disease genetics. (XLS 92 kb)

Additional file 5: Functional enrichment analysis (by WebGestalt) result for each enriched function obtained using levodopa response associated genes. (XLS 48 kb)

Additional file 6: Functional enrichment analysis (by WebGestalt) result for each enriched function obtained using Parkinson’s disease associated genes. (XLS 52 kb)

## Abbreviations

*ABCC8*: ATP binding cassette subfamily C member 8
*ACE2*: Angiotensin I converting enzyme 2
*ACMSD*: Aminocarboxymuconate semialdehyde decarboxylase
*ADORA2A*: Adenosine A2a receptor
ADRs: Adverse drug reactions
AMR: Americans
*ANKK1*: Ankyrin repeat and kinase domain containing 1
*Arf6*: ADP-ribosylation factor 6
*BDNF*: Brain derived neurotrophic factor
BH: Benjamini-Hochberg
*BST1*: Bone marrow stromal cell antigen 1
*CAMK2A*: Calcium/calmodulin-dependent protein kinase II alpha
CAPIT: Core assessment programme for intra-cerebral transplantations
*CCDC62*/*HIP1R*: Coiled-coil domain containing 62
*CCK*: Cholecystokinin
*COMT*: Catechol-O-methyltransferase
*CYP2D6*: Cytochrome P450 family 2 subfamily D member 6
*DDC*: Dopa decarboxylase
*DJ-1*: dj-1 protein
*DRD2*: Dopamine Receptor D2
*DRD3*: Dopamine receptor D3
EAS: East Asians
EUR: European
FDR: False discovery rate
*FGF20*: Fibroblast growth factor 20
*FYN*: FYN proto-oncogene, Src family tyrosine kinase
*GAK*: Cyclin G associated kinase
*GBA*: Glucosylceramidase beta
GO: Gene ontology
*GPNMB*: Glycoprotein nmb
*GRIN2A*: Glutamate ionotropic receptor NMDA type subunit 2A
GRK: G protein-coupled receptor kinase
*GST*: Glutathione S-transferase
GWAS: Genome-wide association study
*HLA-DR*: Major histocompatibility complex, class II, DR
*HOMER1*: Homer scaffolding protein 1
*HTR2A*: 5-hydroxytryptamine receptor 2A
HY: Hoehn and Yahr scale
*IGF1*: Insulin-like Growth Factor 1
*ITGA8*: Integrin subunit alpha 8
LR: Levodopa Response
*LRRK2*: Leucine rich repeat kinase 2
*MAOB*: Monoamine oxidase B
*MAPT*: Microtubule associated protein tau
*MCCC1/LAMP3*: Methylcrotonoyl-CoA carboxylase 1
MeSH: Medical subject headings
*MTHFR*: Methylenetetrahydrofolate reductase
NA: Not applicable
*NAT2*: N-acetyltransferase 2
NR: Not reported
*PARK16*: Parkinson disease 16 (susceptibility)
*PARK2*: Parkinson disease (autosomal recessive, juvenile) 2, parkin
*PARK6*: PTEN induced putative kinase 1
*PARK7*: Parkinsonism associated deglycase
PD: Parkinson’s disease
*PINK1*: PTEN induced putative kinase 1
PKC: Protein kinase C
PPI: Protein-protein interaction
*PRKN*: Parkin RBR E3 ubiquitin protein ligase
*RYR1*: Ryanodine receptor 1
SAS: South Asians
SD: Standard Deviation
*SLC6A3*: Solute carrier family 6 member 3
*SLC6A4*: Solute carrier family 6 member 4
*SNCA*: Synuclein alpha
SNPs: Single nucleotide polymorphisms
*SRC*: SRC proto-oncogene, non-receptor tyrosine kinase
*STK39*: Serine/threonine kinase 39
STR: Short tandem repeat
*SYT11*: Synaptotagmin 11
*TMEM175/DGKQ*/*GAK*: Transmembrane protein 175
tRNA: Transfer RNA
*UBC*: Ubiquitin C
*UCHL1*: Ubiquitin C-terminal hydrolase L1
*UGT1A*: UDP glucuronosyltransferase family 1 member A complex locus
UK BBC: United Kingdom’s brain bank criteria
UK: United Kingdom

## References

[1] Gibrat CS-PM, Bousquet M, Lévesque D, Rouillard C, Cicchetti F (2009). Differences between subacute and chronic MPTP mice models: investigation of dopaminergic neuronal degeneration and alpha-synuclein inclusions. J Neurochem.

[2] Blesa JLJ, Obeso JA (2015). Parkinson's disease: cell vulnerability and disease progression. Front Neuroanat.

[3] J J (2008). Parkinson's disease: clinical features and diagnosis. J Neurol Neurosurg Psychiatry.

[4] Schneider SAOJ (2015). Clinical and pathological features of Parkinson's disease. Curr Top Behav Neurosci.

[5] Group TPS (2004). Levodopa and the progression of Parkinson's disease. N Engl J Med.

[6] Gilgun-Sherki Y, Djaldetti R, Melamed E, Offen D (2004). Polymorphism in candidate genes: implications for the risk and treatment of idiopathic Parkinson's disease. Pharmacogenomics J.

[7] M D, M B, M K (2013). Pharmacogenetics of Parkinson’s disease – through mechanisms of drug actions. Curr Genomics.

[8] Schapira AH, P J (2011). Etiology and pathogenesis of Parkinson’s disease. Mov Disord.

[9] Tan EK (2007). The role of common genetic risk variants in Parkinson disease. Clin Genet.

[10] AB S, Farrer MJ, V B (2013). The genetics of Parkinson's disease: progress and therapeutic implications. Mov Disord.

[11] FK K, McQueen MB, Khoury MJ, Tanzi RE, Bertram L, JP I (2008). Evaluation of the potential excess of statistically significant findings in published genetic association studies: application to Alzheimer's disease. Am J Epidemiol.

[12] Ioannidis JP, Trikalinos TA, MJ K (2006). Implications of small effect sizes of individual genetic variants on the design and interpretation of genetic association studies of complex diseases. Am J Epidemiol.

[13] Little J, Higgins JP, Ioannidis JP, Moher D, Gagnon F, von Elm E, Khoury MJ, Cohen B, Davey-Smith G, Grimshaw J (2009). STrengthening the REporting of genetic association studies (STREGA)--an extension of the STROBE statement. Eur J Clin Investig.

[14] Sagoo GS, Little J, JP H (2009). Systematic reviews of genetic association studies. Human genome epidemiology network. PLoS Med.

[15] Hutton B, Salanti G, Caldwell DM, Chaimani A, Schmid CH, Cameron C, Ioannidis JP, Straus S, Thorlund K, Jansen JP (2015). The PRISMA extension statement for reporting of systematic reviews incorporating network meta-analyses of health care interventions: checklist and explanations. Ann Intern Med.

[16] MEDLINE Database. https://www.ncbi.nlm.nih.gov/pubmed/. Accessed 9 Mar 2016.

[17] Web of Science. http://apps.webofknowledge.com/WOS_GeneralSearch_input.do?%20product=WOS&search_mode=GeneralSearch&SID=X1cFbN52ilAsnVB8QjI&preferencesSaved=. Accessed 9 Mar 2016.

[18] Hewett M OD, Rubin DL, Easton KL, Stuart JM, Altman RB, Klein TE: PharmGKB: the Pharmacogenetics Knowledge Base**.** Nucleic Acids Res 2002 Jan 2002, 30**:**163-165. 10.1093/nar/30.1.163 PMC99138 11752281

[19] Kiferle L, Ceravolo R, Petrozzi L, Rossi C, Frosini D, Rocchi A, Siciliano G, Bonuccelli U, Murri L (2007). Visual hallucinations in Parkinson's disease are not influenced by polymorphisms of serotonin 5-HT2A receptor and transporter genes. Neurosci Lett.

[20] Ivanova SA, Loonen AJ, Pechlivanoglou P, Freidin MB, Al Hadithy AF, Rudikov EV, Zhukova IA, Govorin NV, Sorokina VA, Fedorenko OY (2012). NMDA receptor genotypes associated with the vulnerability to develop dyskinesia. Transl Psychiatry.

[21] Religa D, Czyzewski K, Styczynska M, Peplonska B, Lokk J, Chodakowska-Zebrowska M, Stepien K, Winblad B, Barcikowska M (2006). Hyperhomocysteinemia and methylenetetrahydrofolate reductase polymorphism in patients with Parkinson's disease. Neurosci Lett.

[22] Kaiser R, Hofer A, Grapengiesser A, Gasser T, Kupsch A, Roots I, Brockmoller J (2003). L -dopa-induced adverse effects in PD and dopamine transporter gene polymorphism. Neurology.

[23] Strong JA, Dalvi A, Revilla FJ, Sahay A, Samaha FJ, Welge JA, Gong J, Gartner M, Yue X, L Y (2006). Genotype and smoking history affect risk of levodopa-induced dyskinesias in Parkinson's disease. Mov Disord.

[24] Wells K (2009). Study quality assessment in systematic reviews of research on intervention effects. Res Soc Work Pract.

[25] Szklarczyk D, Franceschini A, Wyder S, Forslund K, Heller D, Huerta-Cepas J, Simonovic M, Roth A, Santos A, Tsafou KP (2015). STRING v10: protein-protein interaction networks, integrated over the tree of life. Nucleic Acids Res.

[26] Wang J, Duncan D, Shi Z, Zhang B (2013). WEB-based GEne SeT AnaLysis toolkit (WebGestalt): update 2013. Nucleic Acids Res.

[27] Kim JS, Kim J-Y, Kim J-M, Kim JW, Chung SJ, Sung R, Kim RN, Mi JK, Kim H-T, Choi K-G, Shin D-I (2011). No correlation between COMT genotype and entacapone benefits in Parkinson’s disease. Neurol Asia.

[28] Schumacher-Schuh AF, Francisconi C, Altmann V, Monte TL, Callegari-Jacques SM, Rieder CR, Hutz MH. Polymorphisms in the dopamine transporter gene are associated with visual hallucinations and levodopa equivalent dose in Brazilians with Parkinson's disease. Int J Neuropsychopharmacol. 2013:1–8. 10.1017/S1461145712001666 23363854

[29] Stefanovic M, Topic E, Ivanisevic AM, Relja M, Korsic M (2000). Genotyping of CYP2D6 in Parkinson's disease. Clin Chem Lab Med.

[30] Fujii C, Harada S, Ohkoshi N, Hayashi A, Yoshizawa K, Ishizuka C, Nakamura T (1999). Association between polymorphism of the cholecystokinin gene and idiopathic Parkinson's disease. Clin Genet.

[31] Yuan RY, Sheu JJ, Yu JM, Hu CJ, Tseng IJ, Ho CS, Yeh CY, Hung YL, Chiang TR (2009). Methylenetetrahydrofolate reductase polymorphisms and plasma homocysteine in levodopa-treated and non-treated Parkinson's disease patients. J Neurol Sci.

[32] Gorgone G, Curro M, Ferlazzo N, Parisi G, Parnetti L, Belcastro V, Tambasco N, Rossi A, Pisani F, Calabresi P (2012). Coenzyme Q10, hyperhomocysteinemia and MTHFR C677T polymorphism in levodopa-treated Parkinson's disease patients. NeuroMolecular Med.

[33] Wu H, Dong F, Wang Y, Xiao Q, Yang Q, Zhao J, Quinn TJ, Chen SD, Liu J (2014). Catechol-O-methyltransferase Val158Met polymorphism: modulation of wearing-off susceptibility in a Chinese cohort of Parkinson's disease. Parkinsonism Relat Disord.

[34] Goetz CG, Burke PF, Leurgans S, Berry-Kravis E, Blasucci LM, Raman R, Zhou L (2001). Genetic variation analysis in parkinson disease patients with and without hallucinations: case-control study. Arch Neurol.

[35] Pascale E, Purcaro C, Passarelli E, Guglielmi R, Vestri AR, Passarelli F, Meco G (2009). Genetic polymorphism of Angiotensin-converting enzyme is not associated with the development of Parkinson's disease and of L-dopa-induced adverse effects. J Neurol Sci.

[36] De Bonis ML, Tessitore A, Pellecchia MT, Longo K, Salvatore A, Russo A, Ingrosso D, Zappia V, Barone P, Galletti P, Tedeschi G (2010). Impaired transmethylation potential in Parkinson's disease patients treated with L-Dopa. Neurosci Lett.

[37] de Lau LM, Verbaan D, Marinus J, Heutink P, van Hilten JJ (2012). Catechol-O-methyltransferase Val158Met and the risk of dyskinesias in Parkinson's disease. Mov Disord.

[38] Paus S, Gadow F, Knapp M, Klein C, Klockgether T, Wullner U (2009). Motor complications in patients form the German competence network on Parkinson's disease and the DRD3 Ser9Gly polymorphism. Mov Disord.

[39] Yahalom G, Kaplan N, Vituri A, Cohen OS, Inzelberg R, Kozlova E, Korczyn AD, Rosset S, Friedman E, Hassin-Baer S (2012). Dyskinesias in patients with Parkinson's disease: effect of the leucine-rich repeat kinase 2 (LRRK2) G2019S mutation. Parkinsonism Relat Disord.

[40] Corvol JC, Bonnet C, Charbonnier-Beaupel F, Bonnet AM, Fievet MH, Bellanger A, Roze E, Meliksetyan G, Ben Djebara M, Hartmann A (2011). The COMT Val158Met polymorphism affects the response to entacapone in Parkinson's disease: a randomized crossover clinical trial. Ann Neurol.

[41] Daniel SE, Lees AJ (1993). Parkinson's disease society brain Bank, London: overview and research. J Neural Transm Suppl.

[42] Gelb DJ, Oliver E, Gilman S (1999). Diagnostic criteria for Parkinson disease. Arch Neurol.

[43] Quinn NBR, Craufurd D, Goldman S, Hodges J, Kieburtz K, Lindvall O, MacMillan J, Roos R (1996). Core assessment program for Intracerebral transplantation in Huntington's disease (CAPIT-HD). Mov Disord.

[44] Cheshire P, Bertram K, Ling H, O'Sullivan SS, Halliday G, McLean C, Bras J, Foltynie T, Storey E, Williams DR (2014). Influence of single nucleotide polymorphisms in COMT, MAO-A and BDNF genes on dyskinesias and levodopa use in Parkinson's disease. Neurodegener Dis.

[45] Foltynie T, Cheeran B, Williams-Gray CH, Edwards MJ, Schneider SA, Weinberger D, Rothwell JC, Barker RA, Bhatia KP (2009). BDNF val66met influences time to onset of levodopa induced dyskinesia in Parkinson's disease. J Neurol Neurosurg Psychiatry.

[46] Greenbaum L, Goldwurm S, Zozulinsky P, Lifschytz T, Cohen OS, Yahalom G, Cilia R, Tesei S, Asselta R, Inzelberg R (2013). Do tardive dyskinesia and L-dopa induced dyskinesia share common genetic risk factors? An exploratory study. J Mol Neurosci.

[47] Ziegler DA, Ashourian P, Wonderlick JS, Sarokhan AK, Prelec D, Scherzer CR, Corkin S (2014). Motor impulsivity in Parkinson disease: associations with COMT and DRD2 polymorphisms. Scand J Psychol.

[48] Tan EK, Cheah SY, Fook-Chong S, Yew K, Chandran VR, Lum SY, Yi Z (2005). Functional COMT variant predicts response to high dose pyridoxine in Parkinson's disease. Am J Med Genet B Neuropsychiatr Genet.

[49] Xie T, Ho SL, Li LS, Ma OC (1997). G/A1947 polymorphism in catechol-O-methyltransferase (COMT) gene in Parkinson's disease. Mov Disord.

[50] Contin M, Martinelli P, Mochi M, Riva R, Albani F, Baruzzi A (2005). Genetic polymorphism of catechol-O-methyltransferase and levodopa pharmacokinetic-pharmacodynamic pattern in patients with Parkinson's disease. Mov Disord.

[51] Liu YZ, Tang BS, Yan XX, Liu J, Ouyang DS, Nie LN, Fan L, Li Z, Ji W, Hu DL (2009). Association of the DRD2 and DRD3 polymorphisms with response to pramipexole in Parkinson's disease patients. Eur J Clin Pharmacol.

[52] Devos D, Lejeune S, Cormier-Dequaire F, Tahiri K, Charbonnier-Beaupel F, Rouaix N, Duhamel A, Sablonniere B, Bonnet AM, Bonnet C (2014). Dopa-decarboxylase gene polymorphisms affect the motor response to L-dopa in Parkinson's disease. Parkinsonism Relat Disord.

[53] Bialecka M, Drozdzik M, Klodowska-Duda G, Honczarenko K, Gawronska-Szklarz B, Opala G, Stankiewicz J (2004). The effect of monoamine oxidase B (MAOB) and catechol-O-methyltransferase (COMT) polymorphisms on levodopa therapy in patients with sporadic Parkinson's disease. Acta Neurol Scand.

[54] Moreau C, Meguig S, Corvol JC, Labreuche J, Vasseur F, Duhamel A, Delval A, Bardyn T, Devedjian JC, Rouaix N (2015). Polymorphism of the dopamine transporter type 1 gene modifies the treatment response in Parkinson's disease. Brain.

[55] Lee MS, Lyoo CH, Ulmanen I, Syvanen AC, Rinne JO (2001). Genotypes of catechol-O-methyltransferase and response to levodopa treatment in patients with Parkinson's disease. Neurosci Lett.

[56] Ward CDGW (1990). Research diagnostic criteria for Parkinson's disease. Adv Neurol.

[57] Oliveri RL, Annesi G, Zappia M, Civitelli D, Montesanti R, Branca D, Nicoletti G, Spadafora P, Pasqua AA, Cittadella R (1999). Dopamine D2 receptor gene polymorphism and the risk of levodopa-induced dyskinesias in PD. Neurology.

[58] Ferrari M, Martignoni E, Blandini F, Riboldazzi G, Bono G, Marino F, Cosentino M (2012). Association of UDP-glucuronosyltransferase 1A9 polymorphisms with adverse reactions to catechol-O-methyltransferase inhibitors in Parkinson's disease patients. Eur J Clin Pharmacol.

[59] Zappia M, Annesi G, Nicoletti G, Arabia G, Annesi F, Messina D, Pugliese P, Spadafora P, Tarantino P, Carrideo S (2005). Sex differences in clinical and genetic determinants of levodopa peak-dose dyskinesias in Parkinson disease: an exploratory study. Arch Neurol.

[60] De Luca V, Annesi G, De Marco EV, de Bartolomeis A, Nicoletti G, Pugliese P, Muscettola G, Barone P, Quattrone A (2009). HOMER1 promoter analysis in Parkinson's disease: association study with psychotic symptoms. Neuropsychobiology.

[61] Acuna G, Foernzler D, Leong D, Rabbia M, Smit R, Dorflinger E, Gasser R, Hoh J, Ott J, Borroni E (2002). Pharmacogenetic analysis of adverse drug effect reveals genetic variant for susceptibility to liver toxicity. Pharmacogenomics J.

[62] Lin JJ, Yueh KC, Lin SZ, Harn HJ, Liu JT (2007). Genetic polymorphism of the angiotensin converting enzyme and L-dopa-induced adverse effects in Parkinson's disease. J Neurol Sci.

[63] Wang J, Liu ZL, Chen B (2001). Dopamine D5 receptor gene polymorphism and the risk of levodopa-induced motor fluctuations in patients with Parkinson's disease. Neurosci Lett.

[64] Schumacher-Schuh AF, Altmann V, Rieck M, Tovo-Rodrigues L, Monte TL, Callegari-Jacques SM, Medeiros MS, Rieder CR, MH H: Association of common genetic variants of HOMER1 gene with levodopa adverse effects in Parkinson's disease patients**.** Pharmacogenomics J 2014, 14:289-294. 10.1038/tpj.2013.37 24126708

[65] Rieck M, Schumacher-Schuh AF, Callegari-Jacques SM, Altmann V, Schneider Medeiros M, Rieder CR, MH H (2015). Is there a role for ADORA2A polymorphisms in levodopa-induced dyskinesia in Parkinson's disease patients?. Pharmacogenomics.

[66] Rieck M, Schumacher-Schuh AF, Altmann V, Francisconi CL, Fagundes PT, Monte TL, Callegari-Jacques SM, Rieder CR, MH H (2012). DRD2 haplotype is associated with dyskinesia induced by levodopa therapy in Parkinson's disease patients. Pharmacogenomics J.

[67] Molchadski I, Korczyn AD, Cohen OS, Katzav A, Nitzan Z, Chapman J, S H-B (2011). The role of apolipoprotein E polymorphisms in levodopa-induced dyskinesia. Acta Neurol Scand.

[68] Kaplan N, Vituri A, Korczyn AD, Cohen OS, Inzelberg R, Yahalom G, Kozlova E, Milgrom R, Laitman Y, Friedman E (2014). Sequence variants in SLC6A3, DRD2, and BDNF genes and time to levodopa-induced dyskinesias in Parkinson's disease. J Mol Neurosci.

[69] Nalls MA, Pankratz N, Lill CM, Do CB, Hernandez DG, Saad M, DeStefano AL, Kara E, Bras J, Sharma M (2014). Large-scale meta-analysis of genome-wide association data identifies six new risk loci for Parkinson's disease. Nat Genet.

[70] Lewis S. Neurodegenerative disorders: Parkinson's disease reveals hidden depths. Nat Rev Neurosci. 2013;14

[71] Dreher J-C KP, Kolachana B, Weinberger DR, Berman KF (2009). Variation in dopamine genes influences responsivity of the human reward system. Proc Natl Acad Sci.

[72] Diatchenko LSG, Nackley AG, Bhalang K, Sigurdsson A, Belfer I, Goldman D, Xu K, Shabalina SA, Shagin D, Max MB, Makarov SS, Maixner W (2005). Genetic basis for individual variations in pain perception and the development of a chronic pain condition. Hum Mol Genet.

[73] Nackley AGSS, Tchivileva IE, Satterfield K, Korchynskyi O, Makarov SS, Maixner W, Diatchenko L (2006). Human catechol-O-methyltransferase haplotypes modulate protein expression by altering mRNA secondary structure. Science.

[74] Habak C, Noreau A, Nagano-Saito A, Mejia-Constain B, Degroot C, Strafella AP, Chouinard S, Lafontaine AL, Rouleau GA, Monchi O (2014). Dopamine transporter SLC6A3 genotype affects cortico-striatal activity of set-shifts in Parkinson's disease. Brain.

[75] Sanz-Blasco S, Avale E, Campana S, Damianich A, Saborido MD, Gomez G, Taravini I, Gershanik O, Ferrario JE. Exploring Fyn as a novel molecule in levodopa induced dyskinesias. Mov Disord. 2015;30

[76] Wang YD, Bao XQ, Xu S, Yu WW, Cao SN, Hu JP, Li Y, Wang XL, Zhang D, Yu SS (2016). A Novel Parkinson's Disease Drug Candidate with Potent Anti-neuroinflammatory Effects through the Src Signaling Pathway. J Med Chem.

[77] Lim KLTJ. Role of the ubiquitin proteasome system in Parkinson's disease. BMC Biochem. 2007;22 10.1186/1471-2091-8-S1-S13 PMC2106364 18047737

[78] Brown AM, Deutch AY, Colbran RJ (2005). Dopamine depletion alters phosphorylation of striatal proteins in a model of Parkinsonism. Eur J Neurosci.

[79] Zhang SF, Xie CL, Wang Q, Liu ZG. Interactions of CaMKII with dopamine D2 receptors: roles in levodopa-induced dyskinesia in 6-hydroxydopamine lesioned Parkinson's rats. Sci Rep. 2015;4(1) 10.1038/srep06811 PMC4212240 25351365

[80] Donaldson JG (2003). Multiple roles for Arf6: sorting, structuring, and signaling at the plasma membrane. J Biol Chem.

[81] Cho DI ZM, Min C, Kwon KJ, Shin CY, Choi HK, Kim KM (2013). ARF6 and GASP-1 are post-endocytic sorting proteins selectively involved in the intracellular trafficking of dopamine D_2_ receptors mediated by GRK and PKC in transfected cells. Br J Pharmacol.

[82] Jan homolak ij, Maša filipovi. The role of IGF-1 in neurodegenerative diseases. Gyrus 2015, 3:162–167.

[83] Felix P, Bernhard SH, Binder G, Weber K, Apel A, Roeben B, Deuschle C, Maechtel M, Heger T, Nussbaum S, Gasser T, Maetzler W, Berg D (2016). Insulin-like growth factor 1 (IGF-1) in Parkinson's disease: potential as trait-, progression- and prediction marker and confounding factors. PLoS One.

[84] Schumacher-Schuh AF, Rieder CR, Hutz MH (2014). Parkinson's disease pharmacogenomics: new findings and perspectives. Pharmacogenomics.

[85] Edwards TL, Scott WK, Almonte C, Burt A, Powell EH, Beecham GW, Wang L, Zuchner S, Konidari I, Wang G (2010). Genome-wide association study confirms SNPs in SNCA and the MAPT region as common risk factors for Parkinson disease. Ann Hum Genet.

[86] Satake W, Nakabayashi Y, Mizuta I, Hirota Y, Ito C, Kubo M, Kawaguchi T, Tsunoda T, Watanabe M, Takeda A (2009). Genome-wide association study identifies common variants at four loci as genetic risk factors for Parkinson's disease. Nat Genet.

[87] Simon-Sanchez J, Schulte C, Bras JM, Sharma M, Gibbs JR, Berg D, Paisan-Ruiz C, Lichtner P, Scholz SW, Hernandez DG (2009). Genome-wide association study reveals genetic risk underlying Parkinson's disease. Nat Genet.

[88] Do CB, Tung JY, Dorfman E, Kiefer AK, Drabant EM, Francke U, Mountain JL, Goldman SM, Tanner CM, Langston JW (2011). Web-based genome-wide association study identifies two novel loci and a substantial genetic component for Parkinson's disease. PLoS Genet.

[89] Hill-Burns EM, Wissemann WT, Hamza TH, Factor SA, Zabetian CP, Payami H (2014). Identification of a novel Parkinson's disease locus via stratified genome-wide association study. BMC Genomics.

[90] Nalls MA, Plagnol V, Hernandez DG, Sharma M, Sheerin UM, Saad M, Simon-Sanchez J, Schulte C, Lesage S, Sveinbjornsdottir S (2011). Imputation of sequence variants for identification of genetic risks for Parkinson's disease: a meta-analysis of genome-wide association studies. Lancet.

[91] Pankratz N, Beecham GW, DeStefano AL, Dawson TM, Doheny KF, Factor SA, Hamza TH, Hung AY, Hyman BT, Ivinson AJ (2012). Meta-analysis of Parkinson's disease: identification of a novel locus, RIT2. Ann Neurol.

[92] Hamza TH, Zabetian CP, Tenesa A, Laederach A, Montimurro J, Yearout D, Kay DM, Doheny KF, Paschall J, Pugh E (2010). Common genetic variation in the HLA region is associated with late-onset sporadic Parkinson's disease. Nat Genet.

[93] Spencer CC, Plagnol V, Strange A, Gardner M, Paisan-Ruiz C, Band G, Barker RA, Bellenguez C, Bhatia K, Blackburn H (2011). Dissection of the genetics of Parkinson's disease identifies an additional association 5′ of SNCA and multiple associated haplotypes at 17q21. Hum Mol Genet.

[94] Vacic V, Ozelius LJ, Clark LN, Bar-Shira A, Gana-Weisz M, Gurevich T, Gusev A, Kedmi M, Kenny EE, Liu X (2014). Genome-wide mapping of IBD segments in an Ashkenazi PD cohort identifies associated haplotypes. Hum Mol Genet.

