# Supplementary material for: A systematic review and integrative approach to decode the common molecular link between levodopa response and Parkinson’s disease
Source: BMC Med Genomics. 2017 Sep 19;10:56. doi: 10.1186/s12920-017-0291-0 (PMC5606117; doi:10.1186/s12920-017-0291-0)
Supplement: Supplementary file 1 — Including Supplementary Material such as Supplementary text S4, S7, Supplementary Tables S5–S6. (DOCX 34 kb) [file 12920_2017_291_MOESM1_ESM.docx]

**An integrative approach to decode molecular link between levodopa response and Parkinson’s disease**

**Online Supplement**

**Supplement s4 (page 8): Selection criteria for the systematic review**

**Supplement s5 (page 9): Methodological Quality assessment of included levodopa induced-ADR studies**

**Supplement s6 (page 9): Methodological Quality assessment of induced levodopa response studies**

**Supplement s-7(page 9): Specification of criteria list for methodological assessment of articles included in systematic review**

**Supplement s4 (page 8): Selection criteria for the systematic review**

**Selection criteria:**

**Inclusion criteria:**

- Studies which includes patients diagnosed with PD by UK Brain Bank Criteria;
- All the case reports, clinical reports, randomized control trials, observational studies and original research articles were included;
- Studies reporting the ethnicity/population of the reported samples;
- Studies reporting association of genetic variants with levodopa alone or with adjuvants;
- Studies that discuss outcome measures evaluating the adverse effect of the administered drug using rating scales like Unified Parkinson’s Disease Rating Scale (UPDRS), Hoehn and Yahr staging scale (HY), Mini-mental state examination (MMSE) or quality of life.

**Exclusion criteria:**

- Articles focused on therapy management;
- Papers including patients suffering from diseases other than PD or presence of any other co-morbid conditions (n= 189);
- Studies based on familial PD (n= 16);
- Studies on in vitro or animal models (n= 76)

**Supplement s5 (page 9): Methodological Quality assessment of included levodopa induced-ADR studies**

| **Criteria** | **1** | **2** | **3** | **4** | **5** | **6** | **7** | **8** | **9** | **10** | **11** | **12** | **13** | **14** | **Cumulative**  **Score** |
| --- | --- | --- | --- | --- | --- | --- | --- | --- | --- | --- | --- | --- | --- | --- | --- |
| **Articles** |  |  |  |  |  |  |  |  |  |  |  |  |  |  |  |
| Schuh A.F.S et al.(2014) | 1 | 1 | 1 | 1 | 1 | 1 | 1 | 1 | 1 | 1 | NA | NA | 1 | 0 | 13 |
| Rieck M., et al (2015) | 1 | 1 | 1 | 1 | 1 | 1 | 1 | 1 | 1 | 1 | NA | NA | 1 | 0 | 13 |
| Strong J.A., et al. (2006) | 1 | 0 | 0 | 1 | 1 | 1 | 0 | 0 | 0 | 1 | NA | NA | 1 | 1 | 9 |
| Rieck M., et.al (2012) | 1 | 1 | 1 | 1 | 1 | 1 | 1 | 0 | 1 | 1 | NA | NA | 1 | 1 | 13 |
| Goldman J. G., et al. (2004) | 1 | 1 | 0 | 1 | 1 | 0 | 0 | 1 | 0 | 0 | NA | NA | 1 | 0 | 8 |
| Oliveri R.L., et al. (1999) | 1 | 1 | 1 | 1 | 1 | 1 | 1 | 1 | 0 | 1 | 1 | 1 | 1 | 1 | 13 |
| Molchadski I., et al.(2011) | 1 | 1 | 1 | 1 | 1 | 1 | 0 | 0 | 0 | 1 | NA | NA | 1 | 1 | 11 |
| Gorgone G., et al (2012) | 1 | 1 | 1 | 1 | 1 | 0 | 1 | 1 | 0 | 1 | NA | NA | 1 | NA | 12 |
| Kaiser R., et al.(2003) | 1 | 1 | 1 | 1 | 1 | 1 | 1 | 1 | 0 | 1 | 1 | 0 | 1 | 1 | 12 |
| Acuña G. et al.(2002) | 1 | 0 | 0 | 0 | 1 | 0 | 0 | 0 | 1 | 0 | NA | NA | 1 | 1 | 7 |
| Pascale E. (2009) | 1 | 1 | 1 | 0 | 1 | 1 | 0 | 0 | 0 | 1 | NA | NA | 1 | 1 | 10 |
| Goetz C.G. et al (2001) | 1 | 1 | 1 | 1 | 0 | 0 | 1 | 0 | 0 | 0 | NA | NA | 1 | 1 | 9 |
| Boutorabi A.T. et al.(2012) | 1 | 1 | 1 | 1 | 1 | 1 | 1 | 1 | 0 | 1 | NA | NA | 1 | 1 | 13 |
| Jee Young Lee(2010) | 1 | 1 | 1 | 1 | 1 | 1 | 1 | 0 | 1 | 1 | NA | NA | 1 | 1 | 13 |
| Foltynie T., et al (2014) | 1 | 1 | 1 | 1 | 1 | 0 | 1 | 1 | 0 | 1 | NA | NA | 1 | 0 | 11 |
| Kiferle L., et al (2007) | 1 | 1 | 1 | 1 | 1 | 1 | 1 | 1 | 1 | 1 | NA | NA | 0 | 1 | 13 |
| Stefanovic M. et al. (2000) | 1 | 0 | 0 | 1 | 1 | 0 | 0 | 0 | 0 | 0 | NA | NA | 0 | 0 | 5 |
| Jian Wang (2001) | 1 | 1 | 1 | 1 | 1 | 1 | 1 | 1 | 0 | 0 | 0 | 0 | 1 | 1 | 10 |
| Lin J.J. et al.(2007) | 1 | 1 | 1 | 0 | 1 | 1 | 1 | 0 | 1 | 1 | NA | NA | 1 | 1 | 12 |
| Religa D (2006) | 1 | 1 | 1 | 1 | 0 | 0 | 1 | 1 | 0 | 0 | NA | NA | 1 | 0 | 9 |
| Maria Luigia De Bonisa(2010) | 1 | 1 | 1 | 1 | 1 | 0 | 1 | 1 | 0 | 0 | NA | NA | 1 | 0 | 10 |
| Schumacher-Schuh AF (2013) | 1 | 1 | 1 | 1 | 1 | 1 | 1 | 1 | 0 | 0 | NA | NA | 1 | NA | 12 |
| Fujii C. et al. (1999) | 1 | 1 | 0 | 1 | 1 | 1 | 0 | 0 | 1 | 1 | NA | NA | 0 | NA | 10 |
| Rey yue Yuan et al. (2009) | 1 | 1 | 1 | 1 | 1 | 1 | 1 | 1 | 0 | 0 | 1 | 1 | 0 | NA | 11 |
| Paus S (2009) | 1 | 1 | 1 | 1 | 1 | 1 | 1 | 1 | 1 | 0 | NA | NA | 1 | 1 | 13 |
| Ziegler DA(2014) | 1 | 1 | 1 | 1 | 1 | 0 | 1 | 1 | 0 | 0 | NA | NA | 1 | 0 | 10 |
| S A Ivanova (2012) | 1 | 0 | 1 | 0 | 0 | 0 | 0 | 0 | 1 | 1 | 1 | 1 | 1 | 0 | 7 |
| Ji Seon Kim et al. (2011) | 1 | 1 | 0 | 1 | 0 | 0 | 1 | 1 | 0 | 1 | 1 | 1 | 1 | 1 | 10 |
| Yahalom G (2012) | 1 | 1 | 1 | 1 | 1 | 1 | 0 | 0 | 1 | 1 | NA | NA | 1 | 0 | 11 |
| De Luca V et al. (2009) | 1 | 1 | 1 | 1 | 1 | 1 | 1 | 1 | 0 | 0 | NA | NA | 1 | 1 | 12 |
| Cheshire P. et al.(2013) | 1 | 1 | 1 | 1 | 0 | 1 | 0 | 0 | 0 | 1 | NA | NA | 1 | 0 | 9 |
| Wu H., et al.(2014) | 1 | 1 | 1 | 0 | 1 | 1 | 0 | 0 | 1 | 0 | NA | NA | 1 | 1 | 10 |
| Corvol J.C., et al. (2011) | 1 | 1 | 0 | 1 | 0 | 0 | 1 | 1 | 0 | 0 | 1 | 1 | 1 | 0 | 8 |
| de Lau L.M. et al. (2012) | 0 | 1 | 0 | 1 | 1 | 1 | 1 | 1 | 0 | 0 | NA | NA | 1 | 1 | 10 |
| Ferrari M., et al. (2012) | 1 | 1 | 1 | 1 | 1 | 0 | 0 | 0 | 0 | 1 | 1 | 0 | 1 | 1 | 9 |
| Zappia M., et al.(2005) | 1 | 1 | 1 | 1 | 1 | 1 | 1 | 0 | 1 | 0 | 1 | 1 | 1 | 1 | 12 |
| Kaplan N., et al.(2014) | 1 | 1 | 1 | 1 | 1 | 1 | 0 | 0 | 1 | 1 | NA | NA | 1 | 0 | 11 |
| Greenbaum L. et. al.(2013) | 1 | 1 | 1 | 0 | 1 | 0 | 1 | 1 | 1 | 1 | NA | NA | 1 | 1 | 12 |

**Supplement s6 (page 9): Methodological Quality assessment of induced levodopa response studies**

| **Criteria** | **1** | **2** | **3** | **4** | **5** | **6** | **7** | **8** | **9** | **10** | **11** | **12** | **13** | **14** | **Cumulative Score** |
| --- | --- | --- | --- | --- | --- | --- | --- | --- | --- | --- | --- | --- | --- | --- | --- |
| **Articles** |  |  |  |  |  |  |  |  |  |  |  |  |  |  |  |
| Tan EK et al. (2005) | 1 | 1 | 1 | 1 | 1 | 0 | 1 | 1 | 0 | 1 | 1 | 1 | 1 | 1 | **12** |
| T. Xie et al. (1997) | NR | 0 | 1 | 1 | 1 | 0 | 0 | 0 | 1 | 0 | NA | NA | NR | 1 | **7** |
| Liu YZ et al. (2009) | 0 | 1 | 1 | 1 | 1 | 1 | 1 | 1 | 0 | 1 | 1 | 1 | 1 | 1 | **12** |
| Devos D et al. (2014) | 1 | 1 | 0 | 1 | 1 | 1 | 1 | 1 | 1 | 1 | NA | NA | 1 | 1 | **13** |
| Moreau C et al. (2015) | 1 | 1 | 1 | 1 | 1 | 0 | 1 | 1 | 1 | 0 | NA | NA | 1 | 1 | **12** |
| Contin M et al. (2005) | 1 | 1 | 1 | 1 | 1 | 0 | 1 | 0 | 1 | 0 | NA | NA | 1 | 1 | **11** |
| Lee MS et al. (2005) | 1 | 1 | 1 | 1 | 1 | 1 | 1 | 1 | 1 | 0 | NA | NA | 1 | 0 | **12** |
| Białecka M et al. (2004) | 1 | 1 | 1 | 1 | 1 | 1 | 1 | 1 | 0 | 0 | NA | NA | NR | 0 | **10** |

**Supplement s7 (page 9): Specification of criteria list for methodological assessment of articles included in systematic review**

**Specification of criteria list for methodological assessmentof articles included in systematic review**

**Study population**

1) Selection of study population- Positive if study population consists of a consecutive sample, assembled at a common early point in the disease.

2) Description of inclusion and exclusion criteria- Positive if criteria are formulated for at least: Number of years since diagnosis/symptoms of Parkinson’s disease,

3) Defined diagnosis of Parkinson’s disease; or in older studies description of relevant signs for the diagnosis (tremor /bradykinesia /rigidity /postural reflex abnormality) combined with the absence of signs (e.g. Babinski sign/Ataxia/early dementia/early autonomic features).

4) Baseline demographic and clinical characteristics are described for all subjects- Positive if age, 5) gender, 6) age at onset, 7) Modified Hoehn and Yahr and UPDRS, 8) at least motor examination, are described.

**Study size**

9) Study size- Positive if the number of patients included in the study is ≥ 100.

**Follow Up**

10) Follow-up ≥ 12 months- Positive if the follow-up period is 12 months or more(modified).

11) Drop-outs/loss to follow-up ≤ 15% or 20%- Positive if total number of drop-outs/loss to follow-up is smaller or equal to 15% with one-year follow-up; and positive if smaller or equal to 20% with two or more years’ follow-up.

12) Information completers versus drop-outs/loss to follow-up- Positive if demographic/clinical information is presented for completers and for drop-outs/loss to follow-up.

**Outcome Measures**

13) Relevance, validity and reproducibility of outcome measures- Positive if at least 1 of the following 4 items are used as outcome measures: impairment, perceived disability in physical activities or performance in physical activities, quality of life, survival and if the study tested the validity/reproducibility of or referred to other studies in which validity/reproducibility of this outcome measure was established.

**Heterogeneity**

14) Heterogeneity is used to describe important differences in studies included in a meta-analysis that may make it inappropriate to combine the studies. Heterogeneity can be clinical (e.g., important differences between study participants, baseline disease severity, and interventions); methodological (e.g., important differences in the design and conduct of the study); or statistical (e.g., important differences in the quantitative results or reported effects)
