# Supplementary material for: A systematic review and integrative approach to decode the common molecular link between levodopa response and Parkinson’s disease
Source: BMC Med Genomics. 2017 Sep 19;10:56. doi: 10.1186/s12920-017-0291-0 (PMC5606117; doi:10.1186/s12920-017-0291-0)
Supplement: Supplementary file 2 — Characteristics of included studies for assessment of association between genetic variants and ADRs in PD (DOCX 123 kb) [file 12920_2017_291_MOESM2_ESM.docx]

**Table 1:** Characteristics of included studies for assessment of association between genetic variants and ADRs in PD

| **Study** | **Population/ Ethnicity** | **Response criteria** | **Age (years)*** | **Gender** | | **Number of samples** | | | **Type of ADR** | **Genes** | **Studied variants** | **p- value** | **OR**  **(95% CI)** | | **Dose* (drug)** | **FP* (years)** | **Score** |
| --- | --- | --- | --- | --- | --- | --- | --- | --- | --- | --- | --- | --- | --- | --- | --- | --- | --- |
|  |  |  |  | **M** | **F** | **Total** | **ADR** | **Non**  **ADR** |  |  |  |  |  |  |  |  |  |
| Schuh A F S et al.[[1](#_ENREF_1)]; t | Brazilian | UPDRS, HY, MMSE | 67.38± 10.34 | 105 | 100 | 205 | 119 | 86 | Motor fluctuations | ***HOMER1*** | rs4704559 | (GG/GA) 0.93 | 0.97 (0.54-1.76)^*^ | | 200 (L) | 1 | 13 |
|  |  |  |  |  |  |  |  |  |  |  | rs10942981 | (GG/GC) 0.68 | 0.88 (0.49-1.61)^*^ | |  |  |  |
|  |  |  |  |  |  |  |  |  |  |  | rs4704560 | (CC/CT) 0.63 | 0.86 (0.47- 1.59)^*^ | |  |  |  |
|  |  |  |  |  |  |  | 86 | 119 | Dyskinesia |  | **rs4704559** | **(GG/GA) 0.04** | **0.53 (0.29-0.98)^*^** | |  |  |  |
|  |  |  |  |  |  |  |  |  |  |  | rs10942981 | (GG/GC) 0.76 | 1.11 (0.61-2.01)^*^ | |  |  |  |
|  |  |  |  |  |  |  |  |  |  |  | rs4704560 | (CC/CT) 0.65 | 0.87 (0.47-1.59)^*^ | |  |  |  |
|  |  |  |  |  |  |  | 54 | 151 | Visual hallucinations |  | rs4704559 | (GG/GA) 0.07 | 0.51(0.25-1.06)^*^ | |  |  |  |
|  |  |  |  |  |  |  |  |  |  |  | rs10942981 | (GG/GC) 0.90 | 1.04 (0.53- 2.05)^*^ | |  |  |  |
|  |  |  |  |  |  |  |  |  |  |  | rs4704560 | (CC/CT) 0.57 | 1.23 (0.61-2.48)^*^ | |  |  |  |
| Rieck M et al.[[2](#_ENREF_2)]; t | Brazilian | UPDRS-IV | ~~63.43 ± 10.39~~  ~~65.06 ± 10.89~~  66.88 ± 10.80 | 110 | 98 | 208 | 90 | 118 | Dyskinesia | ***ADORA2A*** | **rs2298383** | **CT-0.04** | **1.89 (1.03–3.45)** | | 805.14 ± 310.17 (L) | 8.34± 4.86 | 13 |
|  |  |  |  |  |  |  |  |  |  |  |  | **TT-0.02** | **2.06 (1.10–3.82)** | |  |  |  |
|  |  |  |  |  |  |  |  |  |  |  | **rs3761422** | **CC-0.02** | **3.12(1.22-7.96)** | |  |  |  |
|  |  |  |  |  |  |  |  |  |  |  |  | **CT-0.01** | **3.28(1.30-8.27)** | |  |  |  |
|  |  |  |  |  |  |  | 141 | 51 | Motor fluctuations |  | rs2298383 | NA | - | | 754.88 ± 289.09 (L) | 7.36± 4.70 |  |
|  |  |  |  |  |  |  |  |  |  |  | rs3761422 |  | - | |  |  |  |
| Strong J.A et al[[3](#_ENREF_3)]. $ | Caucasian | NR | 65.3±1.56 early; 69.4±1.25 late | 57 | 35 | 92 | 92 | NA | Dyskinesia | *OPRM1* | rs1799971 | 0.06 | 2.8(0.97-4.0) | | NA (L, C) | 5 | 9 |
|  |  |  |  |  |  |  |  |  |  | ***DRD2*** | repeat(13,14) | 0.82 | - | |  |  |  |
|  |  |  |  |  |  |  |  |  |  |  | **14 allele** | **0.04** | **3.4(1.1-10.4)** | |  |  |  |
|  |  |  |  |  |  |  |  |  |  |  | **14/15** | **0.003** | **27.2(1.4-51.0)** | |  |  |  |
| Rieck M et.al[[4](#_ENREF_4)] | Brazilian | UPDRS-IV | 67.08 ± 9.91 | 102 | 97 | 199 | 144 | 55 | Motor fluctuations | *DRD2* | rs1799732 | NA | - | | 759.37 ± 281.85 (L) | 7.65 ± 4.90 | 13 |
|  |  |  |  |  |  |  |  |  |  |  | rs2283265 |  |  |  |  |  |  |
|  |  |  |  |  |  |  |  |  |  |  | rs1076560 |  |  |  |  |  |  |
|  |  |  |  |  |  |  |  |  |  |  | rs6277 |  |  |  |  |  |  |
| **Study** | **Population/ Ethnicity** | **Response criteria** | **Age* (years)** | **Gender** | | **Number of samples**  Table 1: Continued | | | **Type of ADR** | **Genes** | **Studied variants** | **p- value** | **OR**  **(95% CI)** | | **Dose* (drug)** | **FP* (years)** | **Score** |
|  |  |  |  | **M** | **F** | **Total** | **ADR** | **Non**  **ADR** |  |  |  |  |  |  |  |  |  |
|  |  |  |  |  |  |  |  |  |  | *ANKK1* | rs1800497 | NA | - | |  |  |  |
|  |  |  |  |  |  |  |  |  |  |  | rs2734849 |  |  |  |  |  |  |
|  |  |  | 65.52 ± 9.99 |  |  |  | 83 | 116 | Dyskinesia | ***DRD2*** | rs1799732 | - |  |  | 780.12 ± 308.08 (L) | 8.44 ± 4.92 |  |
|  |  |  |  |  |  |  |  |  |  |  | **rs2283265** | **0.05** |  |  |  |  |  |
|  |  |  |  |  |  |  |  |  |  |  | rs1076560 | 0.08 |  |  |  |  |  |
|  |  |  |  |  |  |  |  |  |  |  | rs6277 | - |  |  |  |  |  |
|  |  |  |  |  |  |  |  |  |  | ***ANKK1*** | **rs1800497** | **0.02** |  |  |  |  |  |
|  |  |  |  |  |  |  |  |  |  |  | rs2734849 | - |  |  |  |  |  |
| Goldman J G et al.[[5](#_ENREF_5)] | Chicago | UPDRS, MMSE | 72.1 ± 8.4 | 51 | 37 | 88 | 44 | 44 | Hallucinations | *CCK* | rs1799923 | 0.14 | 0.45 (0.18- 1.11)^*^ | | 655 (L, A, L+A) | NA | 8 |
|  |  |  |  |  |  |  |  |  |  | *CCKAR* | rs1800857 | 0.87 | 1.18(0.50- 2.81)^*^ | |  |  |  |
|  |  |  |  |  |  |  |  |  |  | *CCKBR* | rs1805002 | 0.80 | 0.98 (0.30- 3.15)^*^ | |  |  |  |
| Oliveri R.L et al.[[6](#_ENREF_6)] | Italian | UPDRS-ME, AIMS, MMSE, Hamilton | 64.6 ± 9.4 | 53 | 45 | 98 | 49 | 49 | Dyskinesia | ***DRD2*** | **13** | **0.02** | **0.37(0.15- 0.89)^*^** | | 25mg (C); 250mg (L) | 5 | 13 |
|  |  |  |  |  |  |  |  |  |  |  | **14** | **0.02** | **0.25(0.07- 0.92)^*^** | |  |  |  |
|  |  |  |  |  |  |  |  |  |  |  | **15** | **0.02** | **1.94 (1.08- 3.49)^*^** | |  |  |  |
|  |  |  |  |  |  |  |  |  |  |  | 16 | 0.3 | 1.35 (0.63- 2.89)^*^ | |  |  |  |
|  |  |  |  |  |  |  |  |  |  |  | **13/16** | **0.05** | - | |  |  |  |
|  |  |  |  |  |  |  |  |  |  |  | **15/16** | **0.01** | **3.88 (1.28- 11.74)** | |  |  |  |
| Molchadski I et al.[[7](#_ENREF_7)] | Israeli | NR | 61.1 ± 13 | 90 | 65 | 155 | 89 | 66 | Dyskinesia | *APOE* | Ɛ2 | NA | 1.51 (0.44-5.11)^*^ | | NA (L) | 4.1 ± 3.5 | 11 |
|  |  |  |  |  |  |  |  |  |  |  | Ɛ3 |  |  |  |  |  |  |
|  |  |  |  |  |  |  |  |  |  |  | Ɛ4 |  |  |  |  |  |  |
| Gorgone G et al.[[8](#_ENREF_8)] | Italian | HY | 64.5± 7.7  (cases) | 64 | 78 | 142 | 60 cases | 82 control | Hyper-homocysteinemia | ***MTHFR*** | **rs1801133** | **<0.0001** | **2.59(1.20-5.57)^*^** | | 452.0 ± 170.0 (L) | 1 | 12 |
| Kaiser R et al.[[9](#_ENREF_9)] | Caucasian | UPDRS, HY | 67.0 ± 10.7 | 97 | 74 | 183 | 79 | 93 | Dyskinesia | *DRD2, DRD3* | NA | NA | - | | 386.8 ±103 (L) | 5 | 12 |
|  |  |  | 68.7 ± 10.9 | 99 | 75 |  | 48 | 126 | Psychosis | *DAT* |  |  |  |  | 678.1 ±103 (L) |  |  |
|  |  |  | 65.4 ± 10.8 | 99 | 74 |  | 93 | 80 | Wearing on-off | *NA* |  |  |  |  | 340.8 ±103 (L) |  |  |
| Acuña G et al.[[10](#_ENREF_10)] $ | European | NR | NA | 261 | 148 | 409 | 135 | 274 | Elevated liver | ***UGT1A*** | **C908G** | **0.0018** | - | | NA (T, L) | NA | 7 |
|  |  |  |  |  |  |  |  |  |  |  | **T232G** | **0.01060** |  |  |  |  |  |
| **Study** | **Population/ Ethnicity** | **Response criteria** | **Age* (years)** | **Gender** | | **Number of samples**  Table 1: Continued | | | **Type of ADR** | **Genes** | **Studied variants** | **p- value** | **OR**  **(95% CI)** | | **Dose* (drug)** | **FP* (years)** | **Score** |
|  |  |  |  | **M** | **F** | **Total** | **ADR** | **Non**  **ADR** |  |  |  |  |  |  |  |  |  |
|  |  |  |  |  |  |  |  |  | transaminase levels |  | **A528G** | **0.0008** | - | |  |  |  |
|  |  |  |  |  |  |  |  |  |  |  | **A754G** | **0.0023** |  |  |  |  |  |
|  |  |  |  |  |  |  |  |  |  |  | **A765C** | **0.0023** |  |  |  |  |  |
|  |  |  |  |  |  |  |  |  |  |  | **A197C** | **0.024** |  |  |  |  |  |
|  |  |  |  |  |  |  |  |  |  |  | **G551T** | **0.049** |  |  |  |  |  |
|  |  |  |  |  |  |  |  |  |  |  | **A555C** | **0.0494** |  |  |  |  |  |
|  |  |  |  |  |  |  |  |  |  |  | **A556G** | **0.0494** |  |  |  |  |  |
|  |  |  |  |  |  |  |  |  |  |  | **T786C** | **0.0252** |  |  |  |  |  |
| Pascale E et al.[[11](#_ENREF_11)] | Italian | NR | 60.8±8.1 (age at onset) | NA | NA | 120 cases, 132 control | 45 | 46 | Motor fluctuations | *ACE* | D/I polymorphism | 0.32 | 1.43(0.77-2.65) | | NA (L) | 7.2±4 | 10 |
|  |  |  |  |  |  |  | 33 | 58 | Dyskinesia |  |  | 0.1 | 1.8(0.93-3.95) | |  |  |  |
|  |  |  |  |  |  |  | 15 | 76 | Psychosis |  |  | 0.9 | 0.87(0.38-1.97) | |  |  |  |
| Goetz C G et al.[[12](#_ENREF_12)] € | Chicago, whites | MMSE, UPDRS,  HY, PPRS | 72.1 ± 8.4 | NA | NA | 88 | 44 | 44 | Visual hallucinations | *DRD1* | B1 | 0.27 | - | | 655 ± 326 (L), 2.37 ± 1.70 mg/ day (p) | NA | 9 |
|  |  |  |  |  |  |  |  |  |  |  | B2 | 0.73 |  |  |  |  |  |
|  |  |  |  |  |  |  |  |  |  | *DRD3* | 1 | 0.57 |  |  | 326 (L), 2.37 ± 1.70 |  |  |
|  |  |  |  |  |  |  |  |  |  |  | 2 | 0.43 |  |  |  |  |  |
|  |  |  |  |  |  |  |  |  |  | *APOE* | E2 | 0.06 |  |  |  |  |  |
|  |  |  |  |  |  |  |  |  |  |  | E3 | 0.77 |  |  |  |  |  |
|  |  |  |  |  |  |  |  |  |  |  | E4 | 0.17 |  |  |  |  |  |
| Boutorabi AT et al.[[13](#_ENREF_13)] | Iranian | UPDRS, HY | G1:58.59± 12.3, G2: 56.62± 8.6 | 31 | 72 | 103 | 41 | 62 | Dyskinesia | *COMT* | H allele | 0.66 | 1.07(0.78-1.46) | | 500 (Multiple drugs) | 5 | 13 |
|  |  |  |  |  |  |  |  |  |  | *MAOB* | A allele | 0.74 | 1.15(0.50-2.62) | |  |  |  |
| Lee JY et al.[[14](#_ENREF_14)]; t | Korean | PDSK, DDSK, HY | 62.9±9.3 | 255 | 248 | 503 | 229 | 274 | DDSK and PDSK | *DRD2* | rs1800497 | 0.46 | 1.04(0.67-1.60)^*^ | | NA (L) | ≥5 | 13 |
|  |  |  |  |  |  |  |  |  |  | *DRD3* | rs6280 | 0.63 | 1.76(1.17-2.64)^*^ | |  |  |  |
|  |  |  |  |  |  |  |  |  |  | *GRIN2B* | rs1019385 | 0.53 | 1.16(0.69-1.95)^*^ | |  |  |  |
|  |  |  |  |  |  |  |  |  |  |  | rs7301328 | 0.45 | 1.01(0.65-1.53)^*^ | |  |  |  |
|  |  |  |  |  |  |  |  |  |  |  | rs1806201 | 0.85 | 0.72(0.45-1.17)^*^ | |  |  |  |
|  |  |  |  |  |  |  |  |  |  | *SLC6A4* | 5HTTLPR | 0.76 | 1.04(0.69-1.58)^*^ | |  |  |  |
|  |  |  |  |  | |  | | |  |  |  |  |  | |  |  |  |
| **Study**  Table 1: Continued | **Population/ Ethnicity** | **Response criteria** | **Age* (years)** | **Gender** | | **Number of samples**  Table 1: Continued | | | **Type of ADR** | **Genes** | **Studied variants** | **p- value** | **OR**  **(95% CI)** | | **Dose* (drug)** | **FP* (years)** | **Score** |
|  |  |  |  | **M** | **F** | **Total** | **ADR** | **Non**  **ADR** |  |  |  |  |  |  |  |  |  |
| Foltynie T et al.[[15](#_ENREF_15)];#; t | UK Caucasian | UPDRS | 62.2 | 194 | 121 | 315 | 47 | 268 | Dyskinesia | ***BDNF*** | **rs6265** | **0.001** | 2.12(1.36-3.38) | | NA (L) | 1-2 | 11 |
| Kiferle L et al.[[16](#_ENREF_16)] | Caucasian | UPDRS, MMSE, HY | 62.69±11.52 | 59 | 63 | 312 | 60 | 62 | Visual hallucinations/ Psychosis (psy.) | ***SLC6A4*** | **rs25531** | **>0.01** | **0.86(0.52- 1.44)^*^** | | (L)-259± 117.30 (psy.), 278.2±181.98 (no psy.); (DA) 2.98±1.73 (psy.), 2.78±1.66 (no psy.) | ≥4 | 13 |
|  |  |  |  |  |  |  |  |  |  | ***HTR2A*** | **rs6313** | **>0.05** | **0.94 (0.57- 1.55)^*^** | |  |  |  |
| Stefanovic M et al.[[17](#_ENREF_17)] $ | Croatian | HY (2.5) | 62 | 81 | 105 | 41 case, 145 control | NA | NA | Wearing on- off, Dyskinesia | ***CYP2D6*** | *3, *4, *6, *7, and *8 | **0.03 (*4)** | **2.1(1.11-3.99)** | NA (L) | | NA | 5 |
| Wang J et al.[[18](#_ENREF_18)] β | Chinese | UPDRS III, HY | 60.8±10.0 | 75 | 45 | 120 case, 110 control | 50 (motor) | 50 (non-motor) | Motor fluctuations | *DRD5* | rs6283 ~~T978C~~ | > 0.8 | 1.18(0.71-1.97)^*^ | 250 (L) 25 (C) | | NA | 10 |
| Lin JJ et al.[[19](#_ENREF_19)] | Chinese Taiwanese | UPDRS | NA | 132 | 119 | 251 | 36 | 215 | Dyskinesia | *ACE* | I/D | 0.66 | 1.13(0.67-1.91)^*^ | NA (L) | | 5.0 ± 4.3 | 12 |
|  |  |  |  |  |  |  | 88 | 163 | Wearing on/off |  |  | 0.88 | 1.03(0.70-1.51)^*^ |  |  |  |  |
|  |  |  |  |  |  |  | 63 | 188 | Psychosis |  |  | 0.66 | 1.13(0.67-1.91)^*^ |  |  |  |  |
| Religa D et al.[[20](#_ENREF_20)] # | Caucasian | MMSE, HY (<3) | 70.5±7.57 treated; 66.0±7.11 untreated | NA | NA | 114 case, 100 control | NA | NA | Wearing on-off, hyper-homocystenemia | *MTHFR* | rs1801133 | NA | - | 681.2 ± 328.7 (L) | | NA | 9 |
| De Bonisa ML et al.[[21](#_ENREF_21)] | Italian | UPDRS, HY (1.5-3) | 71.96±4.69 (A1), 65.75±9.60 (A2), | 38 | 18 | 44 (treated) | NA | NA | Hyperhomocysteinemia | ***MTHFR*** | **rs1801133** | **< 0.0001** | **-** | NA (L) | | NA | 10 |
| **Study** | **Population/ Ethnicity** | **Response criteria** | **Age* (years)** | **Gender** | | **Number of samples** | | | **Type of ADR** | **Genes** | **Studied variants** | **p- value** | **OR**  **(95% CI)** | **Dose* (drug)** | | **FP* (years)** | **Score** |
|  |  |  |  | **M** | **F** | **Total** | **ADR** | **Non**  **ADR** |  |  |  |  |  |  |  |  |  |
|  |  |  | 59.93±5.38(B) |  |  | 12 (untreated) |  |  |  |  |  |  |  | NA  (MBI) | |  |  |
| Schuh AFS et al.[[22](#_ENREF_22)] | Brazilian | MMSE, HY | 68.0±10.3 | 100 | 96 | 196 | 50 | 146 | Visual hallucinations | ***DAT1*** | **rs28363170** | **0.02** | **2.5(1.13–5.5)** | 793.2± 409.1 (L) | | > 1 | 12 |
| Fujii C et al.[[23](#_ENREF_23)]; $; a | Japanese | NR | 68.2±9.2 cases, 64.0±9.0 controls | 130 | 81 | 116 case, 95 control | 23 | 93 | Hallucinations | ***CCK*** | 196 G/A, **rs1799923** , 1270 C/G, 6662C/T | **0.02 (rs1799923)** | **0.28 (0.10-0.77)^*^** | 350.4± 140.7 (L) | | 3.9± 4.5 | 10 |
| Yuan RY et al.[[24](#_ENREF_24)] | Taiwanese | HY (1-3) | 71.37± 9.86 | 85 | 101 | 76 cases, 110 control | 48 | 28 | Hyper-homocysteinemia | ***MTHFR*** | **rs1801133 (C677T),** | CC-0.32 **CT-0.004 TT-0.02** | - | 360.21±137.62 (L, A, S/R) | | 6.23 ± 4.33 | 11 |
|  |  |  |  |  |  |  |  |  |  |  | **rs1801131 (A1298C)** | **AA <0.001 AC-0.01** CC-0.17 | - |  |  |  |  |
| Paus S et al.[[25](#_ENREF_25)] | German | HY | 64.7± 10.1 | 364 | 227 | 591 | 117 | 474 | Chorea | ***DRD3*** | **rs6280** | **0.0005** | **-** | NA (L) | | NA | 13 |
|  |  |  |  |  |  |  | 92 | 499 | Dystonia |  |  |  |  |  |  |  |  |
|  |  |  |  |  |  |  | 239 | 352 | Motor complications |  |  | NA |  |  |  |  |  |
| Ziegler D A et al.[[26](#_ENREF_26)] | 122white , 1 Asian | HY-3, MMSE | 66.3±8.7 | 80 | 43 | 123 | NA | NA | Motor complications | *COMT* | rs4680 | NA | - | NA (L, C) | | NA | 10 |
|  |  |  |  |  |  |  |  |  |  | *DRD2* | rs6277 |  |  |  |  |  |  |
|  |  |  |  |  |  |  |  |  |  | *DRD3* | rs6280 |  |  |  |  |  |  |
| Ivanova SA et al.[[27](#_ENREF_27)] $ | Caucasian | AIMS | NA | NA | NA | 143 | 143 | NA | Dyskinesia | ***GRIN2A*** | **~~rs11646587~~** | **~~0.03259~~** |  | NA (L, DA) | | ≥3 | 7 |
|  |  |  |  |  |  |  |  |  |  |  | **rs7192557** | **0.0062** | **3.21(1.37-7.51)** |  |  |  |  |
|  |  |  |  |  |  |  |  |  |  |  | **rs8057394** | **0.0033** | **3.59(1.48-8.71)** |  |  |  |  |
| Ji Seon Kim et al.[[28](#_ENREF_28)] | Korean | UPDRS, HY, ADL | 62.2± 9.2 | NA | NA | 168 | 41 | 127 | Dyskinesia | *COMT* | rs4680 | - | - | 489.3± 176.1(E); 677.9± 270.4 (LEDD) | | 4.9± 3.4 | 10 |
| Yahalom G et al.[[29](#_ENREF_29)] | Isreali (AJ) | NA | 60.6± 13.2 | 211 | 138 | 349 | 190 | 159 | Dyskinesia | *LRRK2* | rs34637584 (carrier) | 0.15 | 1.76(0.83- 3.76)^*^ | NA (L) | | 5.1 ± 5.4 | 11 |
|  |  |  |  |  |  |  |  |  |  |  | rs34637584 (non-carrier) |  |  |  |  | 4.4 ± 4.0 |  |
| **Study** | **Population/ Ethnicity** | **Response criteria** | **Age* (years)** | **Gender** | | **Number of samples** | | | **Type of ADR** | **Genes** | **Studied variants** | **p- value** | **OR**  **(95% CI)** | **Dose* (drug)** | | **FP* (years)** | **Score** |
|  |  |  |  | **M** | **F** | **Total** | **ADR** | **Non**  **ADR** |  |  |  |  |  |  |  |  |  |
| De Luca V et al.[[30](#_ENREF_30)]; t | Southern Italian | UPDRS, HY, MMSE | 70.87± 7.59 | 65 | 66 | 131 | 47 | 84 | Hallucination | ***HOMER1*** | **rs4704559** | **0.004** | **5.89 (1.33- 26.14)^*^** | 676.42 ± 244.38 (L) | | 6 months | 12 |
|  |  |  |  |  |  |  |  |  |  |  | **rs4704560** | **0.04** | **1.79(1.03- 3.10)^*^** |  |  |  |  |
|  |  |  |  |  |  |  |  |  |  |  | rs10942981 | 0.20 | 1.55(0.78-3.06)^*^ |  |  |  |  |
| Cheshire P et al.[[31](#_ENREF_31)]; #; s | UK and Australian | NA | 63.0 ± 9.2 | NA | NA | 285 | NA | NA | Dyskinesia | *COMT* | rs4680 | (Met/ Met) 0.78 | 0.89(0.51- 1.55)^*^ | 794.2 ± 431.6 (L) | | 5 | 9 |
|  |  |  |  |  |  |  |  |  |  | *MAO-A* | rs6323 | 0.12 | - |  |  |  |  |
|  |  |  |  |  |  |  |  |  |  | *BDNF* | rs6265 | (Val/Val) >0.90 | 1.01(0.56- 1.80)^*^ |  |  |  |  |
| Wu H et al.[[32](#_ENREF_32)] | Chinese | NA | NA | 144 | 115 | 516 | 259 cases | 257 control | Wearing off | ***COMT*** | **rs4680** | **GA vs AA-0.01** | **6.54(1.49-28.57)** | 407.45 (Multiple) | | NA | 10 |
|  |  |  |  |  |  |  |  |  |  |  |  | **GG vs AA- <0.001** | **8.84(4.74-16.39)** |  |  |  |  |
| Corvol J C et al.[[33](#_ENREF_33)];$; t | French | UPDRS III, HY | 64 ± 10 | NA | NA | 58 | NA | NA | Motor fluctuations | *COMT* | rs4680 | - | - | 200 (L,E,P) | | NA | 8 |
| de Lau L M et al.[[34](#_ENREF_34)]; t | Dutch | HY, UPDRS, MMSE | 49.9 | 143 | 76 | 219 | 98 | 121 | Dyskinesia | ***COMT*** | **rs4680** | **A allele- 0.004** | **-** | (A-P M) | | NA | 10 |
| Ferrari M et al.[[35](#_ENREF_35)] | Italian | WHO-UMCS | 74.3 ± 6.6 | 37 | 26 | 63 | 11 | 52 | COMT inhibitor induced Toxicity | *UGT1A9* | rs3832043 | 0.81 | 0.79(0.29-2.02) | 554.4 ± 163.5 (E,L) | | 10.3 ± 5.3 | 9 |
|  |  |  |  |  |  |  |  |  |  |  | rs72551330 | 0.09 | 2.87(0.94-8.77) |  |  |  |  |
| Zappia M et al.[[36](#_ENREF_36)] | Italian | UPDRS, HY | 65.2 ± 8.4 | 123 | 92 | 215 | 105 | 110 | Dyskinesia | ***DRD2*** | **13, 14 + CA_n_ STR repeat** | **~~0.010~~**  **0.005** | **0.45(0.26-0.79)** | 654.5 ± 289.6 (L) | | 0.5 | 12 |
| Kaplan N et al.[[37](#_ENREF_37)] | Israeli | NR | 55.2 ±13.5 | 213 | 139 | 352 | 192 | 160 | Dyskinesia | ***SLC6A3*** | **rs393795** | **0.000041** | **4.96(2.3-10.9)** | NA (L) | | 5 ± 4.5 | 11 |
| Greenbaum L et al.[[38](#_ENREF_38)] | Jewish Israeli, Italian | UPDRS | NR | 230 | 160 | 390 | 128 | 75 | Tardive dyskinesia | *HSPG2* | rs2124368 | 0.84, 0 | 1.07(0.54–2.12), - | NA (L) | | ≥3 | 12 |
|  |  |  |  |  |  |  |  |  |  |  | rs2445142 | 0.64,0.92 | 0.88(0.53–1.47), 0.98(0.7–1.36) |  |  |  |  |
|  |  |  |  |  |  |  |  |  |  | *SMYD3* | rs2485914 | 0.9,0.56 | 1.04(0.58–1.83), 1.15(0.73–1.81) |  |  |  |  |
| **Study** | **Population/ Ethnicity** | **Response criteria** | **Age* (years)** | **Gender** | | **Number of samples** | | | **Type of ADR** | **Genes** | **Studied variants** | **p- value** | **OR**  **(95% CI)** | **Dose* (drug)** | | **FP* (years)** | **Score** |
|  |  |  |  | **M** | **F** | **Total** | **ADR** | **Non**  **ADR** |  |  |  |  |  |  |  |  |  |
|  |  |  |  |  |  |  |  |  |  | *GLI2* | rs3943552 | 0.78,0.4 | 0.9(0.43–1.9), 0.79(0.44–1.39) |  | |  |  |
|  |  |  |  |  |  |  |  |  |  | *PCDH7* | rs11728264 | 0.65,0.57 | 0.89(0.56–1.44), 0.9(0.63–1.28) |  |  |  |  |
|  |  |  |  |  |  |  |  |  |  | *DPP6* | rs6977820 | 0.48,0.63 | 1.19(0.73–1.96), 1.08(0.79–1.49) |  |  |  |  |
|  |  |  |  |  |  |  |  |  |  | *KCNB2* | rs4738269 | 0.17,0.43 | 1.67(0.8–3.45), 1.17(0.8–1.7) |  |  |  |  |
|  |  |  |  |  |  |  |  |  |  | ***ABCC8*** | **rs886292** | **0.05**,0.88 | **1.63(1–2.67)**, 1.03(0.75–1.41) |  |  |  |  |
|  |  |  |  |  |  |  |  |  |  | *C14ORF39* | rs4901985 | 0.56,0.73 | 0.81(0.4–1.65), 1.1(0.65–1.86) |  |  |  |  |
|  |  |  |  |  |  |  |  |  |  | *GABRG3* | rs2061051 | 0.68,0.76 | 1.1(0.70–1.71), 0.96(0.7–1.3) |  |  |  |  |
|  |  |  |  |  |  |  |  |  |  | ***RYR1*** | **rs11880894** | 0.26,**0.03** | 0.7(0.39–1.29), **1.26(0.81–1.97)** |  |  |  |  |
|  |  |  |  |  |  |  |  |  |  | *PCSK2* | rs6136064 | 0.42,0.09 | 1.23(0.74–2.05), 0.98(0.71–1.35) |  |  |  |  |
|  |  |  |  |  |  |  |  |  |  | *LARGE* | rs5998941 | 0.15,0.72 | 0.7(0.43–1.14), 0.94(0.68–1.29) |  |  |  |  |
|  |  |  |  |  |  |  |  |  |  | *DRD3* | rs6280 | 0.49,0.14 | 1.18(0.73–1.92), 1.28(0.92–1.78) |  |  |  |  |
|  |  |  |  |  |  |  |  |  |  | *MnSOD* | rs4880 | 0.85,0.98 | 1.04(0.67–1.62), 1(0.74–1.34) |  |  |  |  |
|  |  |  |  |  |  |  |  |  |  | *GSTP1* | rs1695 | 0.99,0 | 0.99(0.59–1.66), - |  |  |  |  |
|  |  |  |  |  |  |  |  |  |  | ***DRD2*** | **rs1800497** | 0.53,**0.04** | 1.25(0.63–2.48), **0.64(0.42–0.98)** |  |  |  |  |
|  |  |  |  |  |  |  |  |  |  | *5HT2A* | rs6313 | 0.71,0.65 | 1.08(0.69–1.7), 0.93(0.68–1.27) |  |  |  |  |
|  |  |  |  |  |  |  |  |  |  | *5HT2A* | rs6311 | 0,0.57 | -, 0.92(0.67–1.24) |  |  |  |  |
|  |  |  |  |  |  |  |  |  |  | *COMT* | rs4680 | 0.3,0.86 | 0.79(0.51–1.23), 1.03(0.75–1.41) |  |  |  |  |
|  |  |  |  |  |  |  |  |  |  | *5HTR2C* | rs6318 | 0.95,0.95 | 1.03(0.53–2.04), 1.02(0.6–1.73) |  | |  |  |

**Table 1: Characteristics of included studies for assessment of association between genetic variants and ADRs in PD.**

Table 1: Continued

M, male; F, female; ADR, Adverse drug reaction; FP, Follow-up Period; AJ, Ashkenazi Jews; UKPDS-BBC,UK Parkinson’s disease society Brain Bank Criteria; UPDRS, Unified Parkinson’s disease rating scale; HY, Hoehn and Yahr Staging of Parkinson's Disease; MMSE, Mini mental state examination; AIMS, Abnormal Involuntary Movement Scale; PPRS, Parkinson’s Psychosis Rating Scale; PDSK,DDSK[[14](#_ENREF_14)]; ADL, Activities of Daily living; WHO-UMC, World health organization-Uppsala Monitoring Centre; PCR-RFLP, Polymerase chain reaction- Restriction fragment length polymorphism; OR, Odds Ratio; CI, Confidence Interval; Drugs are L-levodopa, C-carbidopa, A-amantadine, T-, DA-Dopamine Agonist, MBI-MAO-B inhibitor, S-Selegiline, R-Ropinirole, E-Entacapone, P-Pramipexole, p- pergolide; LEDD, Levodopa equivalent drug dose;

NA, No association, -; Insufficient data.

Score- Cumulative score for Methodological Quality Assessment (Ref **Suppl Table 1a** for detailed scoring)

Odds Ratio, Prevalence Ratio and Hazard Ratio are synonymously used in the table. *OR calculated using reported frequencies from the respective article.

Dose of drug are in mg/day. Unit of Age,

Dose and Follow up period are represented with Mean ± standard deviation;

Greenbaum L et al. two p-values are of Israeli and Italian, respectively.

All the studies recruited PD patients diagnosed by United Kingdom Parkinson’s Disease society brain bank criteria expect $- Not reported, #- by Neurologist/PD Specialist, €- CAPIT, β- Gelb Criteria.

Most of the studies followed PCR-RFLP for genotyping except t- TaqMan, s- Sequenom iPLEXTM, r- RT-PCR, a- ABI PRISM 310.

**Bold** are significant polymorphisms (p≤ 0.05) and their corresponding genes.

References:

1. Schumacher-Schuh AF, Altmann V, Rieck M, Tovo-Rodrigues L, Monte TL, Callegari-Jacques SM, Medeiros MS, Rieder CR, MH H: **Association of common genetic variants of HOMER1 gene with levodopa adverse effects in Parkinson's disease patients.** *Pharmacogenomics J* 2014, **14:**289-294.

2. Rieck M, Schumacher-Schuh AF, Callegari-Jacques SM, Altmann V, Schneider Medeiros M, Rieder CR, MH H: **Is there a role for ADORA2A polymorphisms in levodopa-induced dyskinesia in Parkinson's disease patients?** *Pharmacogenomics* 2015, **16:**573-582.

3. Strong JA, Dalvi A, Revilla FJ, Sahay A, Samaha FJ, Welge JA, Gong J, Gartner M, Yue X, L Y: **Genotype and smoking history affect risk of levodopa-induced dyskinesias in Parkinson's disease.** *Mov Disord* 2006, **21:**654-659.

4. Rieck M, Schumacher-Schuh AF, Altmann V, Francisconi CL, Fagundes PT, Monte TL, Callegari-Jacques SM, Rieder CR, MH. H: **DRD2 haplotype is associated with dyskinesia induced by levodopa therapy in Parkinson's disease patients.** *Pharmacogenomics J* 2012, **13:**1701-1710.

5. Goldman JG, Goetz CG, Berry-Kravis E, Leurgans S, L. Z: **Genetic polymorphisms in Parkinson disease subjects with and without hallucinations: an analysis of the cholecystokinin system.** *Arch Neurol* 2004, **61:**1280-1284.

6. Oliveri RL, Annesi G, Zappia M, Civitelli D, Montesanti R, Branca D, Nicoletti G, Spadafora P, Pasqua AA, Cittadella R, et al: **Dopamine D2 receptor gene polymorphism and the risk of levodopa-induced dyskinesias in PD.** *Neurology* 1999, **53:**1425-1430.

7. Molchadski I, Korczyn AD, Cohen OS, Katzav A, Nitzan Z, Chapman J, S. H-B: **The role of apolipoprotein E polymorphisms in levodopa-induced dyskinesia.** *Acta Neurol Scand* 2011, **123:**117-121.

8. Gorgone G, Curro M, Ferlazzo N, Parisi G, Parnetti L, Belcastro V, Tambasco N, Rossi A, Pisani F, Calabresi P, et al: **Coenzyme Q10, hyperhomocysteinemia and MTHFR C677T polymorphism in levodopa-treated Parkinson's disease patients.** *Neuromolecular Med* 2012, **14:**84-90.

9. Kaiser R, Hofer A, Grapengiesser A, Gasser T, Kupsch A, Roots I, Brockmoller J: **L -dopa-induced adverse effects in PD and dopamine transporter gene polymorphism.** *Neurology* 2003, **60:**1750-1755.

10. Acuna G, Foernzler D, Leong D, Rabbia M, Smit R, Dorflinger E, Gasser R, Hoh J, Ott J, Borroni E, et al: **Pharmacogenetic analysis of adverse drug effect reveals genetic variant for susceptibility to liver toxicity.** *Pharmacogenomics J* 2002, **2:**327-334.

11. Pascale E, Purcaro C, Passarelli E, Guglielmi R, Vestri AR, Passarelli F, Meco G: **Genetic polymorphism of Angiotensin-Converting Enzyme is not associated with the development of Parkinson's disease and of L-dopa-induced adverse effects.** *J Neurol Sci* 2009, **276:**18-21.

12. Goetz CG, Burke PF, Leurgans S, Berry-Kravis E, Blasucci LM, Raman R, Zhou L: **Genetic variation analysis in parkinson disease patients with and without hallucinations: case-control study.** *Arch Neurol* 2001, **58:**209-213.

13. Torkaman-Boutorabi A, Shahidi GA, Choopani S, Rezvani M, Pourkosary K, Golkar M, Zarrindast MR: **The catechol-O-methyltransferase and monoamine oxidase B polymorphisms and levodopa therapy in the Iranian patients with sporadic Parkinson's disease.** *Acta Neurobiol Exp (Wars)* 2012, **72:**272-282.

14. Lee JY, Cho J, Lee EK, Park SS, Jeon BS: **Differential genetic susceptibility in diphasic and peak-dose dyskinesias in Parkinson's disease.** *Mov Disord* 2011, **26:**73-79.

15. Foltynie T, Cheeran B, Williams-Gray CH, Edwards MJ, Schneider SA, Weinberger D, Rothwell JC, Barker RA, Bhatia KP: **BDNF val66met influences time to onset of levodopa induced dyskinesia in Parkinson's disease.** *J Neurol Neurosurg Psychiatry* 2009, **80:**141-144.

16. Kiferle L, Ceravolo R, Petrozzi L, Rossi C, Frosini D, Rocchi A, Siciliano G, Bonuccelli U, Murri L: **Visual hallucinations in Parkinson's disease are not influenced by polymorphisms of serotonin 5-HT2A receptor and transporter genes.** *Neurosci Lett* 2007, **422:**228-231.

17. Stefanovic M, Topic E, Ivanisevic AM, Relja M, Korsic M: **Genotyping of CYP2D6 in Parkinson's disease.** *Clin Chem Lab Med* 2000, **38:**929-934.

18. Wang J, Liu ZL, Chen B: **Dopamine D5 receptor gene polymorphism and the risk of levodopa-induced motor fluctuations in patients with Parkinson's disease.** *Neurosci Lett* 2001, **308:**21-24.

19. Lin JJ, Yueh KC, Lin SZ, Harn HJ, Liu JT: **Genetic polymorphism of the angiotensin converting enzyme and L-dopa-induced adverse effects in Parkinson's disease.** *J Neurol Sci* 2007, **252:**130-134.

20. Religa D, Czyzewski K, Styczynska M, Peplonska B, Lokk J, Chodakowska-Zebrowska M, Stepien K, Winblad B, Barcikowska M: **Hyperhomocysteinemia and methylenetetrahydrofolate reductase polymorphism in patients with Parkinson's disease.** *Neurosci Lett* 2006, **404:**56-60.

21. De Bonis ML, Tessitore A, Pellecchia MT, Longo K, Salvatore A, Russo A, Ingrosso D, Zappia V, Barone P, Galletti P, Tedeschi G: **Impaired transmethylation potential in Parkinson's disease patients treated with L-Dopa.** *Neurosci Lett* 2010, **468:**287-291.

22. Schumacher-Schuh AF, Francisconi C, Altmann V, Monte TL, Callegari-Jacques SM, Rieder CR, Hutz MH: **Polymorphisms in the dopamine transporter gene are associated with visual hallucinations and levodopa equivalent dose in Brazilians with Parkinson's disease.** *Int J Neuropsychopharmacol* 2013**:**1-8.

23. Fujii C, Harada S, Ohkoshi N, Hayashi A, Yoshizawa K, Ishizuka C, Nakamura T: **Association between polymorphism of the cholecystokinin gene and idiopathic Parkinson's disease.** *Clin Genet* 1999, **56:**394-399.

24. Yuan RY, Sheu JJ, Yu JM, Hu CJ, Tseng IJ, Ho CS, Yeh CY, Hung YL, Chiang TR: **Methylenetetrahydrofolate reductase polymorphisms and plasma homocysteine in levodopa-treated and non-treated Parkinson's disease patients.** *J Neurol Sci* 2009, **287:**64-68.

25. Paus S, Gadow F, Knapp M, Klein C, Klockgether T, Wullner U: **Motor complications in patients form the German Competence Network on Parkinson's disease and the DRD3 Ser9Gly polymorphism.** *Mov Disord* 2009, **24:**1080-1084.

26. Ziegler DA, Ashourian P, Wonderlick JS, Sarokhan AK, Prelec D, Scherzer CR, Corkin S: **Motor impulsivity in Parkinson disease: associations with COMT and DRD2 polymorphisms.** *Scand J Psychol* 2014, **55:**278-286.

27. Ivanova SA, Loonen AJ, Pechlivanoglou P, Freidin MB, Al Hadithy AF, Rudikov EV, Zhukova IA, Govorin NV, Sorokina VA, Fedorenko OY, et al: **NMDA receptor genotypes associated with the vulnerability to develop dyskinesia.** *Transl Psychiatry* 2012, **2:**e67.

28. Ji Seon Kim, Ji-Young Kim, Jong-Min Kim, Jae Woo Kim, Sun Ju Chung, Sung R Kim RN, Mi J. Kim, Hee-Tae Kim, Kyoung-Gyu Choi, Dong-Ick Shin, et al: **No correlation between COMT genotype and entacapone beneﬁ ts in Parkinson’s disease.** *Neurology Asia* 2011, **16:**211 – 216.

29. Yahalom G, Kaplan N, Vituri A, Cohen OS, Inzelberg R, Kozlova E, Korczyn AD, Rosset S, Friedman E, Hassin-Baer S: **Dyskinesias in patients with Parkinson's disease: effect of the leucine-rich repeat kinase 2 (LRRK2) G2019S mutation.** *Parkinsonism Relat Disord* 2012, **18:**1039-1041.

30. De Luca V, Annesi G, De Marco EV, de Bartolomeis A, Nicoletti G, Pugliese P, Muscettola G, Barone P, Quattrone A: **HOMER1 promoter analysis in Parkinson's disease: association study with psychotic symptoms.** *Neuropsychobiology* 2009, **59:**239-245.

31. Cheshire P, Bertram K, Ling H, O'Sullivan SS, Halliday G, McLean C, Bras J, Foltynie T, Storey E, Williams DR: **Influence of single nucleotide polymorphisms in COMT, MAO-A and BDNF genes on dyskinesias and levodopa use in Parkinson's disease.** *Neurodegener Dis* 2014, **13:**24-28.

32. Wu H, Dong F, Wang Y, Xiao Q, Yang Q, Zhao J, Quinn TJ, Chen SD, Liu J: **Catechol-O-methyltransferase Val158Met polymorphism: modulation of wearing-off susceptibility in a Chinese cohort of Parkinson's disease.** *Parkinsonism Relat Disord* 2014, **20:**1094-1096.

33. Corvol JC, Bonnet C, Charbonnier-Beaupel F, Bonnet AM, Fievet MH, Bellanger A, Roze E, Meliksetyan G, Ben Djebara M, Hartmann A, et al: **The COMT Val158Met polymorphism affects the response to entacapone in Parkinson's disease: a randomized crossover clinical trial.** *Ann Neurol* 2011, **69:**111-118.

34. de Lau LM, Verbaan D, Marinus J, Heutink P, van Hilten JJ: **Catechol-O-methyltransferase Val158Met and the risk of dyskinesias in Parkinson's disease.** *Mov Disord* 2012, **27:**132-135.

35. Ferrari M, Martignoni E, Blandini F, Riboldazzi G, Bono G, Marino F, Cosentino M: **Association of UDP-glucuronosyltransferase 1A9 polymorphisms with adverse reactions to catechol-O-methyltransferase inhibitors in Parkinson's disease patients.** *Eur J Clin Pharmacol* 2012, **68:**1493-1499.

36. Zappia M, Annesi G, Nicoletti G, Arabia G, Annesi F, Messina D, Pugliese P, Spadafora P, Tarantino P, Carrideo S, et al: **Sex differences in clinical and genetic determinants of levodopa peak-dose dyskinesias in Parkinson disease: an exploratory study.** *Arch Neurol* 2005, **62:**601-605.

37. Kaplan N, Vituri A, Korczyn AD, Cohen OS, Inzelberg R, Yahalom G, Kozlova E, Milgrom R, Laitman Y, Friedman E, et al: **Sequence variants in SLC6A3, DRD2, and BDNF genes and time to levodopa-induced dyskinesias in Parkinson's disease.** *J Mol Neurosci* 2014, **53:**183-188.

38. Greenbaum L, Goldwurm S, Zozulinsky P, Lifschytz T, Cohen OS, Yahalom G, Cilia R, Tesei S, Asselta R, Inzelberg R, et al: **Do tardive dyskinesia and L-dopa induced dyskinesia share common genetic risk factors? An exploratory study.** *J Mol Neurosci* 2013, **51:**380-388.
