# Supplementary material for: A systematic review and integrative approach to decode the common molecular link between levodopa response and Parkinson’s disease
Source: BMC Med Genomics. 2017 Sep 19;10:56. doi: 10.1186/s12920-017-0291-0 (PMC5606117; doi:10.1186/s12920-017-0291-0)
Supplement: Supplementary file 3 — Characteristics of included studies for assessment of association between genetic variants and LR in PD (DOCX 52 kb) [file 12920_2017_291_MOESM3_ESM.docx]

**Table 2:** Characteristics of included studies for assessment of association between genetic variants and LR in PD

| **Study** | **Population/Ethnicity** | **Response criteria** | **Age* (years)** | **Gender** | | **Number of samples** | | | **Genes** | **Studied variants** | **p-value** | **OR (95% CI)** | **Dose* (Drug)** | **Treatment length* (year)** | **Score** |
| --- | --- | --- | --- | --- | --- | --- | --- | --- | --- | --- | --- | --- | --- | --- | --- |
|  |  |  |  | **M** | **F** | **Total** | **R** | **NR** |  |  |  |  |  |  |  |
| Tan EK et al.[[1](#_ENREF_1)] | Singapore | UPDRS | 69.9±7.6 | 24 | 15 | 39 | NA | NA | ***COMT*** | **rs4680** | **0.004** | - | 7.37 mg/ week (P); 421.5 ± 226.2mg (L) | at least 3 months | 12 |
|  |  |  |  |  |  |  |  |  | *DDC* | 4bp del in exon 1 | 0.117 | - |  |  |  |
| Xie T et al.[[2](#_ENREF_2)] | Hong Kong  Chinese | NR | 64.3±9.96 | 31 | 39 | 132 | 70 | 62 | *COMT* | rs4680 | NA | - | NA (L) | NA | 7 |
| Liu YZ et al.[[3](#_ENREF_3)] | Chinese | UPDRS-I, HY <2.5 | 56.80±2.80 | 14 | 16 | 30 | 11 | 19 | *DRD2* | A1/A1 | 0.834 | 1.89( 0.23- 15.74)^*^ | 0.125mg/thrice a day (L, B,P) | > 3 months | 12 |
|  |  |  | 59.60 ± 8.10 |  |  |  |  |  |  | A1/A2 | NA | 0.87( 0.20- 3.90)^*^ |  |  |  |
|  |  |  | 63.00 ± 6.20 |  |  |  |  |  |  | A2/A2 | NA | 0.81( 0.16- 4.20)^*^ |  |  |  |
|  |  |  | 61.90 ± 8.20 |  |  |  |  |  | ***DRD3*** | **rs6280 (Ser/Ser)** | **0.024** | **9.75( 1.60- 59.70)^*^** |  |  |  |
|  |  |  | 57.60 ± 6.50 |  |  |  |  |  |  | Ser/Gly | NA | 0.25( 0.04- 1.46)^*^ |  |  |  |
|  |  |  | 60.50 ± 3.69 |  |  |  |  |  |  | Gly/Gly | NA | - |  |  |  |
| Devos D et al.[[4](#_ENREF_4)] | Caucasian | UPDRS-III, HY | >30 | 23 | 10 | 33 | 14 | 19 | ***DDC*** | **rs921451** | **0.048** | - | NA (L,B) | NA | 13 |
|  |  |  |  |  |  |  | 8 | 25 |  | **rs3837091** |  |  |  |  |  |
| Moreau C et al.[[5](#_ENREF_5)] | French | UPDRS II, III | 60-63 | NA | NA | 61 | NA | NA | ***SLC6A3*** | **rs28363170** | **0.005** | - | 710 ± 90.8mg/day (L) | 16-17 | 12 |
|  |  |  |  |  |  |  |  |  |  | **rs3836790** | **<0.001** | - |  |  |  |
|  |  |  |  |  |  |  |  |  | *DDC* | rs921451 | 0.87 | - |  |  |  |
|  |  |  |  |  |  |  |  |  |  | rs3837091 | 0.7 | - |  |  |  |
|  |  |  |  |  |  |  |  |  | *MAOB* | rs1799836 | 0.4 | - |  |  |  |
|  |  |  |  |  |  |  |  |  | *COMT* | rs4680 | 0.440 | - |  |  |  |
| **Study** | **Population/Ethnicity** | **Response criteria** | **Age (years)** | **Gender** | | **Number of samples** | | | **Genes** | **Studied variants** | **p-value** | **OR (95% CI)** | **Dose* (Drug) (mg/day)** | **Treatment length* (year)** | **Score** |
|  |  |  |  | **M** | **F** | **Total** | **R** | **NR** |  |  |  |  |  |  |  |
| Contin M et al.[[6](#_ENREF_6)] | Italian | HY | 64.3 ± 11.1 | 12 | 7 | 104 | NA | NA | *COMT* | rs4680 | NA | - | 100 mg (L) and 25 mg (B) | 3.0 (0.92 - 8.0) | 11 |
|  |  |  | 61.2 ± 9.10 | 40 | 23 |  |  |  |  |  |  |  |  | 3.0 (0.6 - 5.0) |  |
|  |  |  | 59.1 ± 10.3 | 14 | 8 |  |  |  |  |  |  |  |  | 2.5 (1.0 - 6.0) |  |
| Lee MS et al.[[7](#_ENREF_7)] | Korean | UPDRS | 62 | 33 | 40 | 151 | NA | NA | *COMT* | rs4680 | 0.380 | - | 250mg (L), 25mg (C) | 3.4 | 12 |
| Białecka M et al.[[8](#_ENREF_8)] | Polish | UPDRS, HY | 65.1 ± 9.8 | 55 | 40 | 95 | NA | NA | *COMT* | rs4680 | NA | - | 302.5 ± 99.7 (L) | 5 | 10 |
|  |  |  | 63.8 ± 11.4 |  |  |  |  |  |  |  |  | - | 619.8 ± 138.4 (L) |  |  |

**Table 2: Characteristics of included studies for assessment of association between genetic variants and LR in PD.** M, male; F, female; R, responder; NR, non-responder; bp, base pair; Dose of drug are in mg/day. Unit of Age, Dose and Follow up period are represented with Mean ± standard deviation; UPDRS, Unified Parkinson’s disease rating scale; HY, Hoehn and Yahr Staging of Parkinson's Disease; Drugs are L-levodopa, C-carbidopa, A-amantadine, T-, DA-Dopamine Agonist, MBI-MAO-B inhibitor, S-Selegiline, R-Ropinirole, E-Entacapone, P-Pramipexole; PCR-RFLP, Polymerase chain reaction- Restriction fragment length polymorphism.NA, No Association; -,Insufficient data. **Bold** are significant polymorphisms (p≤ 0.05) and their corresponding genes.

Table 2: Continued

References:

1. Tan EK, Cheah SY, Fook-Chong S, Yew K, Chandran VR, Lum SY, Yi Z: **Functional COMT variant predicts response to high dose pyridoxine in Parkinson's disease.** *Am J Med Genet B Neuropsychiatr Genet* 2005, **137b:**1-4.

2. Xie T, Ho SL, Li LS, Ma OC: **G/A1947 polymorphism in catechol-O-methyltransferase (COMT) gene in Parkinson's disease.** *Mov Disord* 1997, **12:**426-427.

3. Liu YZ, Tang BS, Yan XX, Liu J, Ouyang DS, Nie LN, Fan L, Li Z, Ji W, Hu DL, et al: **Association of the DRD2 and DRD3 polymorphisms with response to pramipexole in Parkinson's disease patients.** *Eur J Clin Pharmacol* 2009, **65:**679-683.

4. Devos D, Lejeune S, Cormier-Dequaire F, Tahiri K, Charbonnier-Beaupel F, Rouaix N, Duhamel A, Sablonniere B, Bonnet AM, Bonnet C, et al: **Dopa-decarboxylase gene polymorphisms affect the motor response to L-dopa in Parkinson's disease.** *Parkinsonism Relat Disord* 2014, **20:**170-175.

5. Moreau C, Meguig S, Corvol JC, Labreuche J, Vasseur F, Duhamel A, Delval A, Bardyn T, Devedjian JC, Rouaix N, et al: **Polymorphism of the dopamine transporter type 1 gene modifies the treatment response in Parkinson's disease.** *Brain* 2015, **138:**1271-1283.

6. Contin M, Martinelli P, Mochi M, Riva R, Albani F, Baruzzi A: **Genetic polymorphism of catechol-O-methyltransferase and levodopa pharmacokinetic-pharmacodynamic pattern in patients with Parkinson's disease.** *Mov Disord* 2005, **20:**734-739.

7. Lee MS, Lyoo CH, Ulmanen I, Syvanen AC, Rinne JO: **Genotypes of catechol-O-methyltransferase and response to levodopa treatment in patients with Parkinson's disease.** *Neurosci Lett* 2001, **298:**131-134.

8. Bialecka M, Drozdzik M, Klodowska-Duda G, Honczarenko K, Gawronska-Szklarz B, Opala G, Stankiewicz J: **The effect of monoamine oxidase B (MAOB) and catechol-O-methyltransferase (COMT) polymorphisms on levodopa therapy in patients with sporadic Parkinson's disease.** *Acta Neurol Scand* 2004, **110:**260-266.
